# *SUGP1* Deficiency Leads to Splicing Dysregulation and Reduced piRNA Abundance in Insects

**DOI:** 10.3390/biology15201852

**Published:** 2026-10-15

**Authors:** Dan Liu, Yongkang Guo, Liting Wang, Mengting Shen, Jing Lv, Peng Wei, Sanyuan Ma, Ling Jia

**Affiliations:** 1 Biological Science Research Center, Southwest University, Chongqing 400716, China; ld1957252393@163.com (D.L.); gyongkang@outlook.com (Y.G.); 13837790647@163.com (M.S.); q10184920@email.swu.edu.cn (J.L.); 2 Key Laboratory of Entomology and Pest Control Engineering, Southwest University, Chongqing 400716, China; wliting01@163.com (L.W.); weipeng2019@swu.edu.cn (P.W.); 3 College of Life Sciences, Guizhou Normal University, Guiyang 550025, China

**Keywords:** *Bombyx mori*, *SUGP1*, piRNA, splicing regulation, Y-box protein, CRISPR/Cas9 editing

## Abstract

SUGP1 (SURP and G-patch domain containing 1) participates in splice-site recognition through its interaction with SF3B1, and loss of this factor leads to aberrant splicing. While its role in pre-mRNA splicing is well-documented, its impact on small RNA pathways remains poorly understood. Here, we used CRISPR/Cas9-mediated *SUGP1* editing in *Bombyx mori* BmE cell populations and RNAi to systemically knock down *SUGP1* in *Drosophila melanogaster*. *SUGP1* loss caused widespread splicing abnormalities in both species and reduced transposon-derived piRNA abundance as shown by small RNA-seq. Notably, the expression and splicing patterns of known piRNA pathway genes remained largely unchanged. Integrating silkworm transcriptomic data with the Flag-SUGP1 Co-IP/MS interactome revealed no direct association with the components of the piRNA biogenesis pathway, but identified a physical interaction with the Y-box protein (YBX), suggesting a potential link connecting splicing surveillance to RNA metabolism and proteostasis. Collectively, these correlative findings suggest that *SUGP1* depletion perturbs global RNA processing, and piRNA reduction may represent a secondary outcome associated with cellular stress or dysregulated RNA metabolism within these two tested insect systems. These results suggest a potential indirect link between *SUGP1*-dependent splicing regulation and piRNA abundance in the tested insect models.

## Simple Summary

Pre-mRNA splicing is a crucial step in eukaryotic gene expression to ensure proper mRNA maturation. This study explores the splicing factor SUGP1, which participates in splice-site recognition and quality control. Although existing research has revealed the important role of SUGP1 in pre-mRNA splicing, its impact on the processing of small RNAs remains unknown. By studying *SUGP1*-targeted silkworm (*Bombyx mori*) cell populations and fruit fly (*Drosophila melanogaster*) lines undergoing systemic RNAi knockdown of *SUGP1*, a decrease in piRNA abundance was observed. To investigate the underlying mechanism, we used the multi-omics approach including transcriptome and protein–protein interaction analysis and revealed that SUGP1 is a spliceosome-associated factor whose loss disrupts global RNA processing rather than directly controlling the known piRNA biogenesis. The study found that when SUGP1 is depleted or functionally perturbed, defective pre-mRNA splicing perturbs RNA processing, coinciding with an activated stress response and concomitant reductions in piRNA abundance. These findings provide insights into how pre-mRNA splicing networks maintain small RNA homeostasis in insect models.

## 1. Introduction

In eukaryotes, pre-mRNA splicing removes intronic sequences from nascent transcripts, and this process must be executed with high precision to maintain the integrity of the proteome [1,2,3]. SUGP1 (SURP and G-patch domain containing 1) helps to recognize the branch point during the early assembly process of the spliceosome and participates in a quality control step to distinguish between correct and cryptic splice sites [4,5,6]. Its dysfunction causes widespread aberrant splicing [6,7,8,9,10]. However, beyond canonical pre-mRNA splicing, whether SUGP1 extends its regulatory reach to small non-coding RNA pathways remains poorly understood.

Genome-wide screens in *Drosophila melanogaster* (*D. melanogaster*) and *Caenorhabditis elegans* (*C. elegans*) have consistently identified many spliceosomal components as potential regulators of PIWI-interacting RNA (piRNA) biogenesis (e.g., *CG9213*, *RnpS1*, *CG2931*, *Acn* in *D. melanogaster*; *prp-21, rsp-7*, *smu-2*, *prp-4*, *uaf-2* in *C. elegans*) [11,12,13,14]. Splicing factors repeatedly appear in these screening experiments, which raises an unresolved question: are these proteins genuinely participating in piRNA processing acting as “moonlighting” factors, or is their apparent involvement an indirect consequence of maintaining overall RNA metabolic health? SUGP1 occupies a relatively specialized position within the spliceosome. Rather than contributing to catalysis per se, it carries out branch-point proofreading and G-domain-mediated monitoring of splice-site choice. This distinction makes SUGP1 a useful entry point for separating direct from indirect effects. In key insect models like *B. mori* and *D. melanogaster*, the connection between core splicing factors and piRNA biogenesis has received little experimental attention, and clarifying whether *SUGP1* loss affects piRNA biogenesis through direct or indirect mechanisms would help fill this gap.

PIWI-interacting RNAs (piRNAs) are a class of highly abundant single-stranded non-coding RNAs with a size distribution of 24–32 nucleotides (nt) that associate with PIWI subfamily proteins [15,16,17]. A large number of mature piRNAs map to transposable elements (TEs) and serve as sequence-specific guides to repress transposon activity and promote the degradation of their transcripts, thereby maintaining genomic and cellular homeostasis [18,19,20,21,22]. piRNAs originate from heterochromatic regions known as piRNA clusters. These genomic reservoirs can be classified into uni-strand and dual-strand clusters according to their transcription patterns. This difference in origin directly determines the initiation mode of their initial biogenesis [17,20]. Two tightly coupled pathways govern piRNA biogenesis: the Primary Pathway and the Secondary Pathway [23,24]. After cluster-derived precursor transcripts are exported and processed by cytoplasmic endonucleases and modification factors [25,26,27,28,29], primary piRNAs typically display a pronounced 5′ uridine (1U) bias [19,24]. These primary complexes then enter the ping-pong cycle catalyzed by their respective PIWI protein partners (such as *Siwi* and *BmAgo3* in *B. mori*) [30,31,32]. Through a reciprocal feedback loop, this cycle achieves exponential amplification of the silencing signal and imparts two molecular signatures on secondary piRNAs: a 5′ uridine preference (1U) and an adenine at the 10th position (10A) [19,24,33], with the assistance of specialized helicases and cofactors [34,35,36,37].

In this study, we exploited the unique advantages of the silkworm as a research model: it has a fully functional piRNA mechanism (including primary and secondary pathways) [23,38,39], a highly diverse piRNA library in its compact genome [15], and a well-established genome-wide CRISPR knockout system, which facilitates efficient functional validation [40]. We used CRISPR/Cas9 technology to construct *SUGP1*-KO silkworm cell populations and RNAi technology to generate *Drosophila SUGP1* knockdown (SUGP1-KD) strains. We adopted an integrative omics strategy: RNA-seq was utilized to capture transcriptomic alterations in splicing and expression, while Co-Immunoprecipitation Mass Spectrometry (Co-IP/MS) in silkworm cells was performed to map the potential SUGP1 interactome. By combining direct molecular interaction assays with global expression profiling analysis, our goal is to clarify whether SUGP1 directly regulates the piRNA biosynthesis mechanism or indirectly affects piRNA abundance through broader perturbations in RNA processing. We also verify whether the candidates identified in the interactome, such as the multi-functional RNA-binding protein Y-box protein (YBX) [41,42,43,44], provide a regulatory link between splicing surveillance, downstream RNA metabolism, and piRNA homeostasis in insect models.

## 2. Materials and Methods

### 2.1. The Design of sgRNA and Components of Knockout Experiments

Gene sequence information was retrieved from the silkDB3.0 database (https://silkdb.bioinfotoolkits.net/; accessed 14 October 2026). The gene IDs are as follows: *SUGP1* (KWMTBOMO12558), *Y-box protein* (KWMTBOMO00339), *Hen1* (KWMTBOMO08756). The sgRNA for single gene knockout was designed using the CCTop online tool (https://cctop.cos.uni-heidelberg.de/, accessed 14 October 2026). NGG was selected as the PAM sequence, AAGT was used for the 5′ end joint of the sense strand, and AAAC was used for the 5′ end joint of the antisense strand. The seed sequence consisted of 12 bases, with a maximum of 2 mismatched bases allowed. Silkworm (*B. mori*) was selected as the target species for prediction. To ensure the selection of sgRNAs with high credibility from the CCTop output results, we made the final selection based on the sgRNA scores and rankings provided by this tool and considered the following factors: (1) selecting sgRNAs with low off-target activity (minimizing off-target sites, especially in exonic regions), (2) selecting sgRNAs with high rankings (scores greater than or equal to 0.74), and (3) avoiding sgRNAs with consecutive identical nucleotides in the final selected sequence. To maximize knockout efficiency, sgRNAs were prioritized within the first exon near the 5′ terminus using the CCTop tool (https://cctop.cos.uni-heidelberg.de/, accessed 14 October 2026). When the candidate sequences in the initial exon failed to meet the strict design criteria, the search was sequentially extended to the second and third exons. The sgRNA targeting sequences of both *SUGP1* and *Hen1* are located in the first exon. The sgRNA sequences targeting the target genes and the predicted off-target sites in *B. mori* genome are summarized in Supplementary Table S1. The off-target sites were predicted by CCTop online tool across the entire genome. The off-target effects were checked by RNA-seq alignment (IGV) and verified by Sanger sequencing, and no obvious non-specific off-target effects were detected.

For this study, we used the IE2-spCas9 from the Biological Science Research Center of Southwest University as the nuclease in the CRISPR/Cas9 system [45]. The piggyBac vector (from Biological Science Research Center, Southwest University, Chongqing, China), harboring the U6-sgRNA-scaffold module, served as the target recognition element [40]. The sgRNA designed by CCTop was synthesized in the form of primers. The F and R primers (each at a working concentration of 10 µM) were mixed in equal volumes in nuclease-free water (without additional annealing buffer), denatured at 98 °C for 7 min, and then wrapped in cotton to allow gradual cooling down to room temperature for double-strand annealing. The piggyBac vector was digested with *AarI* (Cat. No. ER1581, Thermo Scientific™, Waltham, MA, USA). The digested backbone and the annealed sgRNA product were ligated using T4 DNA ligase (Cat. No. FL101-01, TransGen Biotech, Beijing, China) to generate the recombinant sgRNA expression plasmids.

To systematically validate the editing efficiency and specificity in each independent experiment, cells were harvested around 5 days after transfection and used as a mixed population (polyclonal pool). Genomic DNA was extracted from these edited polyclonal cell populations, and genomic regions flanking the sgRNA cleavage site were PCR-amplified using specific primers designed by Primer Premier 5 (Premier Biosoft, Palo Alto, CA, USA; targeting a region approximately 1000 bp upstream and downstream). The obtained PCR products were subjected to Sanger sequencing to characterize the editing events. In the early stage of omics sequencing, we confirmed the target disruption in the *SUGP1*-edited polyclonal cell populations through genomic PCR coupled with Sanger sequencing (ICE deconvolution; https://ice.editco.bio/; accessed 14 October 2026) and subsequent verification of targeted transcript-level disruptions across the cleavage site in RNA-seq alignments using IGV. The estimated editing efficiencies ranged from 13% to 28% across the tested silkworm cell populations, and the main mutation types were frameshift deletion mutations (mainly deletions of −6 bp, −5 bp, and −1 bp).

### 2.2. Plasmid Construction and Molecular Cloning

The CDS template of *SUGP1* was derived from the cDNA of *B. mori* BmE cells. The specific primer sequences are located in the Supplementary Materials (Supplementary Table S1). All primers used in this study were synthesized by Tsingke Biotechnology (Chongqing, China) and purified via polyacrylamide gel electrophoresis (PAGE). The Flag-SUGP1 overexpression vector was constructed using the laboratory-modified *B. mori* Bm1180 vector (from Biological Science Research Center, Southwest University) [46,47].

The CDS fragment of *SUGP1* was amplified using the cDNA of *B. mori* BmE cells as a template, *EGFP* was amplified from the piggyBac vector (from Biological Science Research Center, Southwest University [40]), and IE2 was amplified from the IE2-spCas9 plasmid (from Biological Science Research Center, Southwest University [45]). Flag-tag sequence was added to the amplification primers of SUGP1/EGFP and IE2 in the form of homologous arms, and IE2-Flag-SUGP1 and IE2-Flag-EGFP were connected into a fragment by overlap extension PCR. The linearized vector backbone was prepared by digesting the custom vector backbone Bm1180 with *SalI* and *NotI* (Cat. No: *SalI* = R0138V, *NotI* = R0189V, New England Biolabs, Ipswich, MA, USA), followed by gel purification. The high-fidelity 2x Hieff Canace^®^ PCR Master Mix High Fidelity Enzyme Premix (Cat. No. 10149ES01, Yeasen Biotechnology, Shanghai, China) was used to amplify the insert sequence “IE2-Flag-SUGP1” with specific primers containing 15–25 bp homologous sequences corresponding to the ends of the linearized vector. According to the manufacturer’s instructions, the target fragment was seamlessly assembled into the Bm1180 backbone using the homologous recombination cloning kit (Cat. No. CU201-02, TransGen Biotech, Beijing, China).

The sgRNA expression vectors targeting *SUGP1*, *YBX* and *Hen1* were constructed utilizing a laboratory-modified piggyBac backbone, which harbors a U6 promoter and a down-stream sgRNA scaffold. The sgRNA insertion site on this vector is flanked by two inversely oriented recognition sites for *AarI* (Cat. No. ER1581, Thermo Scientific™), a Type IIS restriction endonuclease. The empty vector was digested with *AarI* to generate a linearized backbone with specific non-palindromic sticky ends. Concurrently, the designed forward and reverse sgRNA oligonucleotides were synthesized and annealed to form double-stranded DNA inserts with compatible overhanging ends. Finally, the annealed double-stranded sgRNAs were seamlessly inserted into the *AarI*-digested piggyBac vector using T4 DNA ligase (Cat. No. FL101-01, TransGen Biotech, Beijing, China), yielding the complete circular sgRNA expression plasmids designed for targeted genomic editing. Transfection of these sgRNA vectors yielded polyclonal cell pools with partial editing efficiencies of 13–28%. Thus, all subsequent molecular and phenotypic readouts represent mixed cell populations harboring residual unedited alleles, rather than complete gene loss at the whole-population level.

Target fragments were recovered using the Gel Extraction Kit (Omega Biotek, Norcross, GA, USA). Transformation was performed using Trans-T1 competent cells (TransGen Biotech, Beijing, China). Endotoxin-free plasmid extraction was conducted with the QIAprep Spin Mini Prep Kit (QIAGEN, Shanghai, China).

### 2.3. Culture Methods

The BmE cell line (derived from embryos) and BmN cell line (derived from ovary) were obtained from the Biological Science Research Center, Southwest University. Preliminary experiments confirmed that *B. mori* cells possess a complete piRNA biogenesis pathway [38]. Cells were passaged every two days by replacing the medium with Grace (BmE cells)/TC100 (BmN cells) insect culture medium supplemented with 10% fetal bovine serum (Gibco, Thermo Fisher Scientific, Waltham, MA, USA), with an average passage interval of 2 days.

The insect cell culture media were prepared in the laboratory using commercial Grace’s Insect Medium basal powders (unsupplemented; Cat. No. 11300027, Thermo Fisher Scientific, Waltham, MA, USA). The formulation consisted of the reconstituted basal powder supplemented with 3.0 g/L lactalbumin hydrolysate, 0.35 g/L sodium bicarbonate, 2% (*v*/*v*) penicillin-streptomycin. TC-100 Insect Medium was prepared from powder (Product No. T2000, USBiological, Salem, MA, USA) with 2% (*v*/*v*) penicillin-streptomycin. Both media were adjusted to pH 6.27–6.40 and filter-sterilized before use.

Both BmE and BmN cells were initially transfected with the targeted editing sgRNA vectors. The target genomic region was amplified by PCR, followed by Sanger sequencing and visualized alignment using the Integrative Genomics Viewer (IGV, v2.19.7). Combined with the observation of cell viability phenotypes, the targeted genomic disruption and editing efficiency at the target locus were evaluated.

### 2.4. Transfection of BmE/BmN Cells

Healthy BmE or BmN cells at 80–90% confluency were seeded into 6-well plates (Corning® CellBIND®; Corning, Corning, NY, USA) at roughly 60% density and allowed to attach overnight. Each transfection well received 3.2 µg of plasmid DNA and 8 µL of Roche X-treme GENE HP reagent (Roche X-treme GENE HP DNA Transfection Reagent; Roche, Basel, Switzerland). The reagent was first diluted in 200 µL of Grace’s Insect Medium, while the plasmid was separately diluted in another 200 µL of the same medium. After combining the two dilutions, the mixture sat at room temperature for 20 min to allow complex formation, then was added dropwise to the wells. Incubation proceeded at 27 °C for 6–8 h before the medium was replaced with fresh pre-warmed medium. The specific culture time is related to the type of experiment: 48–72 h for protein expression assays, or approximately 5 days for CRISPR-mediated knockout experiments.

### 2.5. Cell Viability Assay

The Cell Counting Kit-8 (CCK-8) reagent was purchased from Beyotime Biotechnology (Shanghai, China; Cat. No. C0038). In a 12-well plate, the old medium was aspirated and each well washed once using 250 µL PBS. Then, 500 µL of medium (Grace’s medium for BmE cells or TC-100 medium for BmN cells, supplemented with 10% FBS, as described in the “Section 2.3”) and 50 µL of CCK8 reagent (10:1) were added to the wells in the dark. The plate was incubated at 27 °C for 3–5 h, and absorbance was measured at 450 nm. The cell survival rate was calculated as: Cell survival rate = [(As − Ab)/(Ac − Ab)] × 100% (As: OD value of the experimental well, Ac: OD value of the control well, Ab: OD value of the blank well).

For the CCK-8 assay, the experiment was performed with three independent biological replicates (*n* = 3), and each replicate was from a separate transfection experiment conducted on a different date. For each biological replicate, three technical parallel wells were measured and the mean value was calculated to minimize the effects of pipetting and instrumental variation.

### 2.6. Drosophila RNAi Lines and Sample Collection

The RNA interference (RNAi) line targeting *SUGP1* (VDRC ID: V25779) and control (V60000) were obtained from the Vienna *Drosophila* Resource Center (VDRC, Vienna, Austria). To achieve systemic knockdown of *SUGP1*, the UAS-RNAi line was crossed with a ubiquitous *Gal4* driver (*act5C*-*Gal4*/CyO, Tsinghua University, Beijing, China; Stock #00033). All flies were reared on standard cornmeal-yeast-agar medium at 25 °C under a 12 h light/dark cycle.

The standard *Drosophila* medium was prepared in the laboratory. Specifically, the medium was composed of 9.2 g/L soybean powder, 66.83 g/L cornstarch, 25 g/L yeast powder, 40 g/L sucrose, 42.4 g/L maltose, and 6 g/L agar in ultrapure water. After boiling, 1 g/L sodium benzoate, 6.88 mL/L propionic acid, and 0.9 g/L methyl p-hydroxybenzoate (pre-dissolved in ethanol) were added to the medium prior to dispensing it into culture tubes [48].

For both high-throughput sequencing and quantitative expression analyses, three completely independent biological replicates were established. Each biological replicate consisted of a synchronized sample pool of 10 3-day-old adult *Drosophila* (5 males and 5 females) collected from independent culture bottles. *Drosophila* samples represent a composite of various somatic and germline tissues, which is different from the homogeneous silkworm cell culture model. Therefore, they capture the comprehensive responses at the organism-level rather than cell-autonomous effects. To verify knockdown efficiency, total RNA was extracted from these pooled whole-body tissues using TRIzol reagent (Thermo Fisher Scientific, Waltham, MA, USA). RT-qPCR assays were performed to determine the relative mRNA expression of *SUGP1*, utilizing *Tub80B* as an internal reference. A knockdown was considered effective and validated when a statistically significant decrease in *SUGP1* expression (*p* < 0.05, determined by Student’s *t*-test) was observed relative to the control group. Only these validated *Drosophila* cohorts were utilized for subsequent sequencing and experiments.

### 2.7. RNA-Seq

High-throughput mRNA sequencing analyses were performed using independent biological replicate samples, which were derived from different batches of silkworm cells or *Drosophila* populations treated in different weeks. Total RNA was extracted from the samples (Thermo Fisher Scientific, Waltham, MA, USA; Catalog No. 10296010) and digested with DNaseI (Thermo Fisher, Waltham, MA, USA). RNA purity and concentration were assessed using the Agilent 2100 Bioanalyzer (Agilent Technologies, Santa Clara, CA, USA) and NanoDrop UV spectrophotometer (Thermo Fisher Scientific, Waltham, MA, USA). After confirming RNA quality met the requirements (RNA integrity was assessed using the Agilent 2100 Bioanalyzer, and samples with RIN > 8 and concentration > 20 ng/µL were used for library construction), library construction was performed using Oligo(dT) magnetic beads and hexamer random primers. The insert fragment size (approximately 300 bp) was verified using the Agilent 2100 Bioanalyzer, and library concentration was quantified with the ABI StepOnePlus Real-Time PCR System (Applied Biosystems, Waltham, MA, USA, Catalog No. 4376600). Sequencing was performed using the MGI T7 sequencer (MGI, Shenzhen, China). RNA sequencing was performed by Qiantang Biotechnology Co., Ltd. (Suzhou, China).

RNA-seq reads were processed with nf-core/rnaseq (v3.23.0) using Trim Galore (v0.6.10) for adapter/quality trimming, STAR 2.7.11b for species-specific genome alignment, and Salmon (v1.10.3) for gene-level abundance estimation. For *B. mori,* reads were aligned to the NCBI reference genome assembly ASM3026992v2 (accession: GCF_030269925.1) using the chromosome-level sequence FASTA and genomic_chr.gtf annotation file. For *D. melanogaster*, reads were mapped to the dm6 reference genome based on FlyBase release r6.67 using dmel_r6_67.fa and dmel-all-r6.67.stranded.gtf.gz. MultiQC (v1.33) was used to summarize QC and mapping statistics. Detailed RNA-seq quality and alignment metrics (raw reads, clean reads, overall mapping rate, uniquely mapped reads, unique mapping rate, and biological replicate correlation coefficients) for each library have been comprehensively recorded in Supplementary File S1 (Quality_metrics_and_data_deposition: “RNA-seq quality metrics” sheet). Differential expression was performed with nf-core/differentialabundance (v1.5.0) using DESeq2 (v1.34.0) on gene-level counts between the KO/KD group and the WT group. DESeq2 size-factor normalization used the median-of-ratios method (sf_type = ratio), Wald tests were used for contrasts, and *p* values were adjusted by the Benjamini–Hochberg method. Parameters used in the nf-core/rnaseq pipeline can be referred to https://gitee.com/luxiangze/bm_pirna/blob/main/workflow/RNA-seq_workflow/nf-params.json (accessed 14 October 2026). Parameters used in the nf-core/differentialabundance pipeline can be referred to https://gitee.com/luxiangze/bm_pirna/blob/main/workflow/RNAdiff_workflow_ncbi_20260311/nf-params.json (accessed 14 October 2026).

Alternative splicing was analyzed with a Snakemake workflow (v2.7.8a). Paired-end reads were aligned with STAR 2.7.11b, BAM files were grouped by condition, and rMATS v4.1.2 was run with paired-end mode, strand-specific library setting ‘fr-firststrand’, variable-read-length mode, and read length 150. The workflow generated both junction-count-only (JC) and junction-count-plus-exon-count (JCEC) rMATS tables for five event classes: skipped exon (SE), retained intron (RI), mutually exclusive exons (MXE), alternative 5′ splice site (A5SS), and alternative 3′ splice site (A3SS). The primary summary and volcano plot used JC results to focus on splice-junction-supported events. Selecting JC results is to conduct a more conservative and direct measurement of alternative splicing and avoid potential quantitative biases introduced by uneven read coverage within exons. It is worth noting that parallel analysis using JCEC results yielded highly consistent splicing patterns and exactly the same core conclusions. Significant events were defined as events with rMATS FDR < 0.05. The inclusion-level difference (IncLevelDifference, |ΔPSI| ≥ 0.05) was used to indicate the direction and magnitude of splicing change. Representative significant events were detected using RT-PCR (Supplementary File S3: Uncropped_original_gel_images). All code and parameters were stored in https://gitee.com/luxiangze/bm_pirna/tree/main/workflow/rnasplicing (accessed 14 October 2026).

### 2.8. Small RNA-Seq

Total RNA was extracted using the MJZol RNA Extraction Kit (Majorbio, Shanghai, China). Small RNA libraries (16–35 nt) were constructed using the QIAseq miRNA Library Kit (Qiagen, Hilden, Germany), and sequencing was performed with the SE75 mode on the Illumina NovaSeq Xplus platform (Illumina, San Diego, CA, USA). Small RNA sequencing was conducted by Qiantang Biotechnology Co., Ltd. (Suzhou, China). The raw sequencing datasets (including both RNA-seq and small RNA-seq) have been deposited in the NCBI Sequence Read Archive (SRA) database under the BioProject accession number PRJNA1337577.

Small RNA reads were processed with FastQC (v0.12.1), Cutadapt (v5.2), SeqKit (v2.12.0), and Bowtie (v1.3.1). Quality control and removal of 3′ adapters from the raw smRNA-seq data were performed using Cutadapt (v5.2) with the following parameters: -q 20 -m 18 -M 37 -j 16 -a AACTGTAGGCACCATCAAT --discard-untrimmed. Contaminants (miRNA, rRNA, tRNA, snoRNA, and snRNA) were removed using Bowtie (v1.3.1). The reads were then filtered to retain those with lengths of 24–35 nt (for *B. mori*) or 23–35 nt (for *D. melanogaster)* using Seqkit (v2.12.0). The total count of these size-selected, non-structural reads served as the effective library size for downstream RPM normalization, following established literature [49]. Among these size-selected, non-structural reads, only those successfully aligned by Bowtie (v1.3.1) to the reference transposable element databases—comprising previously annotated elements in a previous study [50] and seven additional sequences from NCBI (Kabuki, AB032718.1; Yamato, AB042122.1; Bm1-Neet, AB455915.1; Matsui, AB455919.1; Pao, L09635.1; mag, X17219.1; and Noguchi, AB455904.1)—were defined as transposable element-derived piRNAs. The small RNAs of 23–35 nt in *Drosophila* were aligned to the transposon library annotated by FlyBase (dmel_r6.67_FB2026_01). One mismatch was allowed, and the alignment parameters were -p 16 -v 1 -q -S --best --al. The molecular identity of these reads as authentic piRNAs was further validated by assessing the 1U/10A nucleotide bias and characteristic 10-nt ping-pong overlap signatures. Comprehensive small RNA-seq sequencing and processing metrics for all samples—including raw reads, clean reads, size-filtered reads (24–35 nt for *B. mori* and 23–35 nt for *D. melanogaster*), structural RNA-filtered reads, transposable element (TE)-mapped reads, and pairwise biological replicate correlation coefficients—are summarized in the Supplementary File S1 (Quality_metrics_and_data_deposition: ‘small RNA-seq quality metrics’ sheet).

To quantify and compare the abundances of piRNAs between the control group and the SUGP1-perturbed groups, referring to the methods in published literature [49], the alignment read counts of transposable element-derived piRNAs were normalized to reads per million (RPM) based on the total non-structural small RNA library size. Statistical evaluation of small RNA-seq data from three biological replicate samples (*n* = 3) were performed at multiple structural levels using GraphPad Prism (v8.0.2): For the length distribution profiles and the abundance at the transposable element (TE) class-level abundances, multiple unpaired two-tailed Student’s *t*-tests were used to analyze the differences in the normalized reads per million mapped reads (RPM) values for each size window or major transposable subcategory without assuming equal variances (using Welch’s correction). To control for multiple testing, the two-stage linear step-up procedure of Benjamini, Krieger and Yekutieli (BKY method) was used to control the FDR. An FDR threshold of <0.05 was set to indicate statistical significance. At the granular level of individual transposon families, the same framework of multiple *t*-tests corrected by BKY FDR was applied to identify differentially represented elements. All code related to Small RNA-seq data analysis is hosted at https://gitee.com/luxiangze/bm_pirna/tree/main/bm_pirna/smrna_seq (accessed 14 October 2026).

### 2.9. Real-Time Fluorescence Quantitative PCR (RT-qPCR)

Total RNA was extracted using the HiPure Universal RNA Mini Kit (Magen Bio, Guangzhou, China). RNA reverse transcription was performed using the One-Step gDNA Removal and cDNA Synthesis SuperMix (TransGen Biotech, Beijing, China). Quantitative PCR (qPCR) was conducted using Top Green qPCR SuperMix (+Dye I; TransGen Biotech, Beijing, China). All qPCR primers were designed using Premier 5 software (Premier Biosoft, Palo Alto, CA, USA). The size of the optimized amplicons was 100–200 bp, with a balanced GC content (40–60%), and the tendency for primer secondary structure or primer-dimer formation was minimized. The specificity of the primers was experimentally verified by 1.5% agarose gel electrophoresis. The results showed that only a single clear product of the expected size appeared, and no non-specific products or primer dimers were detected. Gene expression was quantified relative to the internal reference gene *Bmrp49* in silkworm, *Tub80B* was used as the internal reference for *D. melanogaster* RT-qPCR assays. Relative gene expression levels were calculated using the cycle threshold (Ct) method with the reference gene as a control: ΔCt = Ct(target gene) − Ct(reference gene). To ensure technical consistency and minimise the effects of pipetting errors, at least three technical replicate measurements (*N* ≥ 3) were simultaneously performed on each of the three independent biological replicate samples. Primer sequences for the genes are provided in the Supplementary Table S1. Quantitative real-time PCR (RT-qPCR) assays were performed using a qTOWER^3^ G Real-Time PCR System (Analytik Jena, Jena, Germany).

### 2.10. Co-Immunoprecipitation (Co-IP)

After transfecting *B. mori* BmE cells with the Flag-SUGP1 overexpression vector for 48 h, the cells were harvested and lysed on ice for 30 min with 300 µL (per well of a 6-well plate) of NP40 lysis buffer (Beyotime Biotechnology, Shanghai, China) containing 1× protease inhibitor cocktail. After centrifugation at 12,000× *g* for 15 min at 4 °C, the total cellular proteins (protein samples) were collected from the supernatant.

To identify protein interaction factors, Co-IP and subsequent mass spectrometry experiments were performed, and three independent biological replicates were set up (*n* = 3). Normal mouse immunoglobulin G (IgG) (Beyotime Biotechnology, Shanghai, China) and Flag-EGFP were used as negative controls to remove non-specific background binders. Briefly, 40 µL of Dynabeads™ Protein G (ThermoFisher, Waltham, MA, USA) were mixed and washed in a rotator with 100 µL of NP40 lysis buffer containing protease inhibitor (Beyotime, Shanghai, China) (100:1) for 1 min per wash. The beads were then incubated with 100 µL of NP40 buffer and antibody/protease inhibitor (control IgG: 2–4 µL, typically 3 µL) for 30–40 min. After washing with NP40 buffer containing protease inhibitor, 200 µL of crosslinking agent (Reagent configuration ratio: 1 mL conjugation buffer (2.4 g/L NaH_2_PO_4_, 8.8 g/L NaCl, adjusted to pH 7.9 and filtered) + 0.0029 g BS3 (a crosslinker that covalently stabilizes weak or transient receptor-ligand interactions; Cat. No. 21580, Thermo Scientific Pierce, Waltham, MA, USA) was added, and the mixture was incubated for 30–40 min at room temperature on a rotator. Following this, 12.5 µL of quenching buffer (1 M Tris-HCl) was added, and incubation continued for 15 min. After discarding the supernatant, the beads were washed with PBS and then incubated with the protein sample (approximately 600 μg) at 4 °C for 6 h with gentle rotation. The supernatant was discarded, and the beads were washed with PBS. For protein sample preparation, the bead-bound immunoprecipitated complexes were eluted by the addition of 100–120 μL of SDT elution buffer (4% SDS in 50 mM Tris-HCl). Following a 5 min incubation at room temperature, the beads were separated using a magnetic rack, the supernatant containing the eluted proteins was carefully collected. The eluted protein samples were subsequently mixed with 5× SDS protein loading buffer and denatured by heating at 95 °C for 10 min before downstream applications.

### 2.11. Mass Spectrometry (MS)

For each sample, the above immunoprecipitated protein eluate was directly processed using a 30 kDa molecular weight cut-off centrifugal filter (Sartorius, Göttingen, Germany) by the filter-aided sample preparation (FASP) method, without prior SDS-PAGE fractionation. After on-filter digestion with sequencing-grade trypsin (Promega, Madison, WI, USA), the resulting peptides were analyzed on an Orbitrap Fusion Lumos mass spectrometer coupled to an EASY-nLC 1200 system (Thermo Fisher Scientific, Waltham, MA, USA) operated in data-dependent Top-Speed mode. Raw spectra were searched with MaxQuant (v2.1.3.0) against a composite database of the *B. mori* NCBI reference proteome (NCBI-Bombyx_mori-35439+4.fasta) and project-specific entries (R24111.fasta), using trypsin digestion (≤2 missed cleavages), carbamidomethylation (C) as fixed and oxidation (M)/N-terminal acetylation/phospho-STY as variable modifications, with both PSM and protein FDR controlled at 1% and label-free quantification (LFQ + iBAQ); LC-MS/MS analysis was performed by Shanghai Omicsspace Biotech Co., Ltd (Shanghai, China). The confidence level of protein identification is evaluated using the Andromeda score generated by the integrated Andromeda search engine. The Andromeda peptide score is calculated based on the matching between experimental and theoretical tandem mass spectra using a binomial probability model (expressed as −10 × log_10_P), and the corresponding protein score is the sum of the best peptide scores of the unique peptides and razor peptides of that protein. A higher Andromeda score indicates higher sequence coverage, better spectral matching quality, and higher identification confidence.

To define bona fide SUGP1-interacting proteins with high stringency, a qualitative presence/absence classification strategy relative to the negative control (IgG) was adopted: Candidate proteins must have ≥1 unique peptide; Candidate proteins should show reproducible identification results in the Flag-SUGP1 group (MS count ≥ 1 in each replicate sample and LFQ intensity > 0); Candidate proteins should be completely absent in the corresponding IgG negative control (peptide count = 0, MS count = 0, and LFQ intensity = 0); Potential contaminants, reverse decoy-matched proteins, and proteins identified only by sites were excluded. Since the baseline counts and intensities in the IgG control were undetectable (0), the candidate interacting proteins represent a qualitative, all-or-none specific enrichment rather than a continuous fold-change threshold screening. It should be noted that the Flag-EGFP control was only used as the experimental negative control for Western blotting and silver staining to verify the specificity of immunoprecipitation, and was not included in the subsequent mass spectrometry filtering pipeline. The identification of candidate interacting proteins in the mass spectrometry results was performed with reference to the matched IgG negative control.

### 2.12. Western Blot (WB)

Cultured cells were collected and washed twice with PBS. NP40 lysis buffer and protease inhibitor mixture (Beyotime Biotechnology, Shanghai, China) were mixed at a 100:1 ratio and added to the cell samples. The samples were gently mixed and incubated on ice for 30 min. After centrifugation at 12,000 rpm for 15 min at 4 °C, the supernatant was collected as the total protein extract. Protein concentration was determined using the BCA Protein Quantification Kit (Beyotime Biotechnology, Shanghai, China). Equal amounts of protein were mixed with 5 × SDS-PAGE loading buffer (Beyotime Biotechnology, Shanghai, China) and heated at 95 °C for 10 min. SDS-PAGE (4.5% stacking gel and a 12.5% separating gel; Cat. No. PG113, Epizyme, Shanghai, China) was performed, followed by wet transfer of the proteins to a PVDF membrane (0.22 μm; Cat. No. E802, Vazyme, Nanjing, China). The membrane was blocked with 5% skim milk and incubated at room temperature for 1 h with primary antibodies (1:5000 dilution) against HA (rabbit monoclonal; abclonal, Shanghai, China), Flag (mouse monoclonal; HUABIO, Hangzhou, China), and tubulin (mouse monoclonal; Beyotime, Shanghai, China). After washing, the membrane was incubated with a 1:5000 dilution of HRP-conjugated secondary antibody (goat anti-mouse/rabbit IgG; Beyotime, Shanghai, China) at room temperature for 1 h. Immuno-reactive products were visualized using the Enhanced ECL Chemiluminescent Substrate Kit (Yeasen Biotechnology, Shanghai, China).

### 2.13. Immunofluorescence (IF)

The culture medium was discarded, and cells were washed with PBS. Cells were fixed with 4% paraformaldehyde (PFA; Sigma-Aldrich, St. Louis, MO, USA) for 15 min, followed by washes with pre-cooled PBS. Permeabilization was performed with 0.3% Triton™ X-100 (Sigma-Aldrich, St. Louis, MO, USA) for 10 min, and then cells were incubated with 5% goat serum (Beyotime, Shanghai, China) at 37 °C for 30 min. After removing the blocking solution, cells were incubated overnight at 4 °C with primary antibodies (rabbit anti-HA; Abclonal, Waltham, MA, USA, and mouse anti-Flag; Huabio, Hangzhou, China) diluted in PBS. The next day, the antibody solution was removed, and cells were washed with PBS. Fluorescent secondary antibodies (Cy3-conjugated goat anti-mouse IgG (H + L) and FITC-conjugated goat anti-rabbit IgG (H + L); Beyotime, Shanghai, China) were added and incubated in the dark at 37 °C for 1 h. Nuclei were stained with DAPI (1 µg/mL; Beyotime, Shanghai, China), and images were acquired using a confocal microscope.

Fluorescence images were acquired using a Zeiss LSM 880 confocal laser scanning microscope (Carl Zeiss, Oberkochen, Germany) equipped with a 40×/1.4 NA oil-immersion objective. Samples were excited using 405, 488, and 561 nm laser lines. Image acquisition and processing were performed with ZEN software (v2.3; Carl Zeiss, Oberkochen, Germany). All immunofluorescence assays were independently repeated in three biological batches (*n* = 3). For each condition, at least 5 non-overlapping fields of view were acquired in both the panoramic overview and magnified details, and more than 30 co-transfected dual-positive cells were evaluated to confirm the reproducibility of the observed subcellular distribution (additional representative fields of view and magnifications are provided in the Supplementary Materials). The ImageJ software (ImageJ 1.54g; NIH, Bethesda, MD, USA) was used to plot the line-scanning fluorescence intensity curves of single representative cells (RGB analysis) and Manders’ coefficient to quantitatively evaluate the consistency of the spatial signals.

### 2.14. Silver Staining

Following SDS-PAGE, protein products were visualized using a Fast Silver Staining Kit (Cat. No. P0017S, Beyotime, Shanghai, China) according to the manufacturer’s protocol. All necessary reagents, including the silver solution and the STOP solution, were provided within the kit. The gel was placed into 100 mL fixed solution (50 mL ethanol, 10 mL acetic acid and 40 mL Milli-Q pure water (Millipore, Burlington, MA, USA)), and shaken for 40 min at room temperature on a 60–70 rpm shaker (SK series; Zhejiang Lichen Instrument Technology Co., Ltd., Shaoxing, China). After fixation, the gel was shaken with 30% ethanol at 60–70 rpm for 10 min, followed by washing with 200 mL Milli-Q water for 10 min. The water was discarded, and 100 mL of silver dye sensitizer solution (1×) was added, with shaking at 60–70 rpm for 2 min. The gel was then washed with 200 mL Milli-Q water. Next, 100 mL of silver solution (1×) was added, and the gel was shaken at 60–70 rpm for 10 min. Finally, 100 mL of the prepared silver staining solution was added, and the gel was shaken at room temperature for 3–10 min until the protein products were visible. The silver staining solution was discarded, and 100 mL of 1× silver staining stop solution was added, followed by shaking at room temperature for 10 min. After color development, the stop solution was discarded, and the gel was washed with 100 mL Milli-Q water, shaking for 5 min.

### 2.15. Data Analysis and Visualization

Fluorescence intensity, length (cell diameter), and other measurements were analyzed using ImageJ, and data visualization was performed with GraphPad Prism. Images related to alternative splicing variability and analyses of piRNA derived from transposons were generated using custom-programmed R scripts hosted (https://gitee.com/luxiangze/bm_pirna; accessed 14 October 2026).

Differential gene expression was analyzed with DESeq2. *p* values were adjusted for multiple testing using the Benjamini–Hochberg procedure, and DEGs were defined as genes with FDR (padj) < 0.05 and |log2FoldChange| > 1. Alternative splicing was analyzed with rMATS, and significant splicing events were defined as events with rMATS FDR < 0.05. For small RNA analyses, read abundances were normalized as RPM. The small RNA length distribution, nucleotide-bias, piRNA abundance, and transposon-derived piRNAs analyses were RPM-based analyses. For comparisons between two independent groups, a two-tailed Student’s *t*-test was employed. For comparisons between multiple experimental groups and a single control group, Welch’s one-way analysis of variance (ANOVA) followed by Dunnett’s T3 post hoc test was used.

The orthologous information of *B. mori* and *D. melanogaster* was obtained from OrthoDB v12. We used shell scripts to extract 43,009 shared orthologous groups (OGs) containing both species (taxon IDs 7091 and 7227) from odb12v2_OG2genes. Subsequently, by combining the odb12v2_genes table and the FlyBase annotation set, the internal numbers in OrthoDB were mapped to the standard NCBI GeneID and Drosophila FlyBase ID, respectively. Finally, a Python (v3.10.12) script was used to flatten the data into all orthologous gene pairs and complete the classification and annotation of the preset gene subsets.

Visualization of results, including piRNA positional nucleotide biases (1U/10A) and splicing events of known piRNA biogenesis pathway genes, was implemented using custom computational pipelines in https://gitee.com/luxiangze/bm_pirna/tree/main/R_scripts (accessed 14 October 2026).

## 3. Results

### 3.1. Depletion of SUGP1 Induces Similar Splicing Dysregulation in B. mori and D. melanogaster

**Figure 1:**
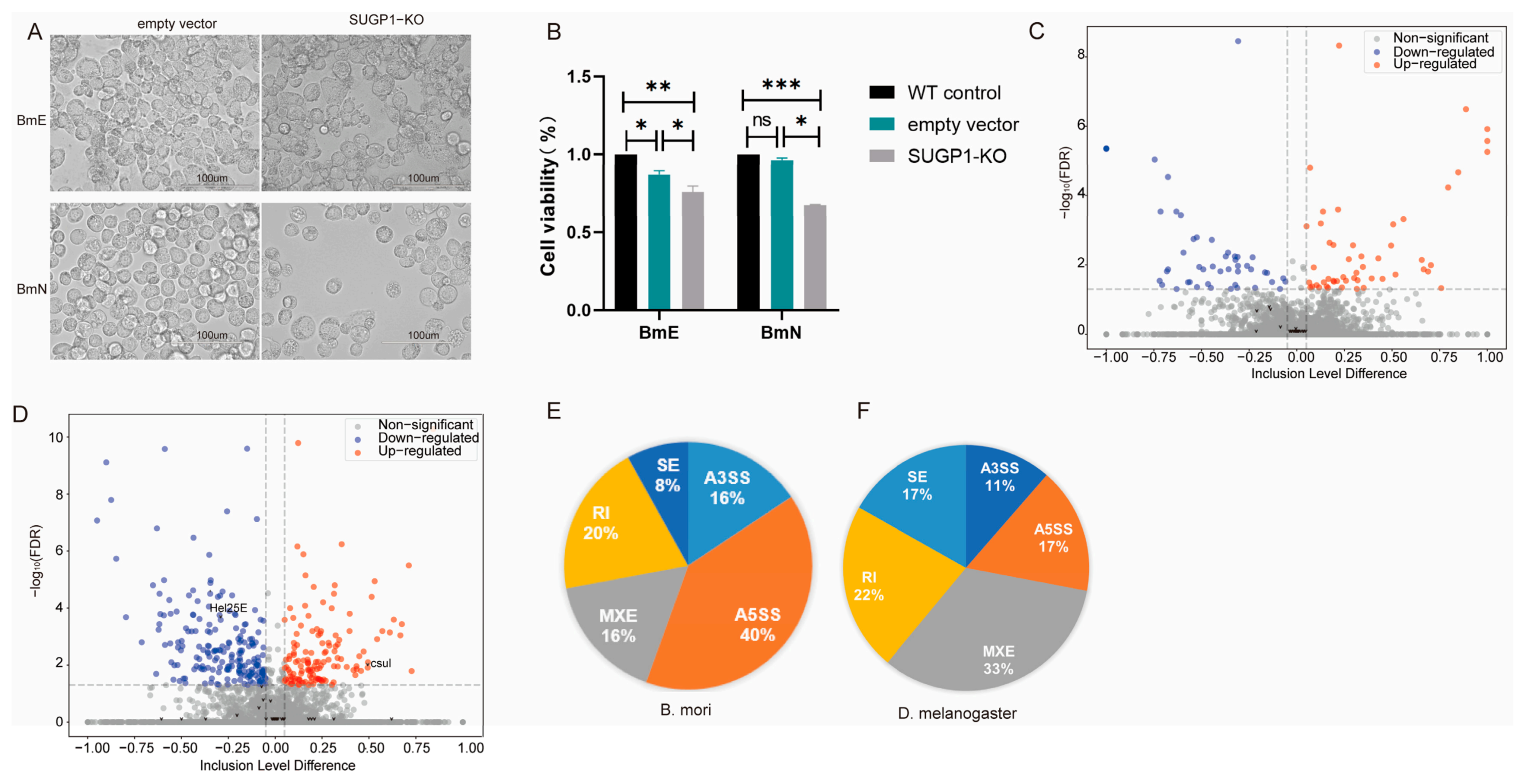
Depletion of SUGP1 induces similar splicing dysregulation in *B. mori* and *D. melanogaster*. (**A**) Representative morphological images of BmE (upper) and BmN (lower) cells following *SUGP1* knockout (*SUGP1*-KO) using sgRNA1 (TCTTGTACTCAAAGCAGATT targeting exon 1). Control cells (left) were transfected with an empty vector (no gene editing of *SUGP1*). *SUGP1*-KO polyclonal cell populations (right) exhibit pronounced vacuolation and decreased density. Scale bars, 100 μm. (**B**) Relative cell viability following *SUGP1*-KO (5 days post-transfection). Both wild-type (WT) and empty-vector-transfected cells served as controls, and values are normalized to the WT control. Pairwise statistical comparisons among all three groups (WT vs. EV, EV vs. *SUGP1*-KO, and WT vs. *SUGP1*-KO) are explicitly indicated by comparison brackets above the bars. Data are presented as mean ± SD (derived from three independent biological replicates (*n* = 3)). Statistical significance was determined using Welch’s one-way ANOVA followed by Dunnett’s T3 post hoc test (ns, not significant; * *p* < 0.05; ** *p* < 0.01; *** *p* < 0.001). (**C**,**D**) Alternative splicing events upon SUGP1 perturbation in polyclonal *B. mori* BmE cell populations (*SUGP1*-KO, (**C**)) and 3-day-old adult *D. melanogaster* pools (5 males and 5 females per replicate; SUGP1-KD, (**D**)). The *X*-axis shows the difference in splicing inclusion (ΔPSI, Percent Spliced In, quantifying the relative inclusion ratio of alternative exons) between *SUGP1*-KO/KD and the WT control with vertical dashed lines demarcating the cutoff threshold of |ΔPSI| ≥ 0.05, and the *Y*-axis represents -log_10_(FDR) (False Discovery Rate) calculated by rMATS, with the horizontal dashed line indicating the significance cutoff of FDR = 0.05 (-log10(FDR) = 1.30). Each circle symbol represents an alternative splicing event: orange and blue circles represent significantly up-regulated and down-regulated splicing events, respectively (|ΔPSI| ≥ 0.05, FDR < 0.05), while gray circles denote non-significant events. Known genes of piRNA biogenesis pathway are indicated by black arrows (most located within the gray dot cluster). (**E**,**F**) Proportion of major alternative splicing changes (significant events were first quantified by alternative splicing type and then summarized to calculate the final overall proportion), including exon skipping (SE), alternative 5′ splice site (A5SS), alternative 3′ splice site (A3SS), retained intron (RI), and mutually exclusive exons (MXE).

SUGP1 serves as a pivotal regulator of RNA processing and stability [4,5,6]. To determine its biological role in insects, we first employed the CRISPR/Cas9-mediated gene knockout (KO) to deplete *SUGP1* in *B. mori* BmE and BmN cells. Six gRNAs targeting exons 1 (*n* = 3) and 3 (*n* = 3) were designed and screened; the most efficient sgRNA1 (TCTTGTACTCAAAGCAGATT) was subsequently used to generate *SUGP1*-KO polyclonal cell populations in both BmE and BmN cells, yielding an estimated editing efficiency of 25–28% (Figure S1A,B,D). *SUGP1*-KO polyclonal cells showed clear phenotypic deterioration relative to the control: pronounced vacuolization, reduced cell density, and significantly lower viability (Figure 1A,B). These observations suggest that SUGP1 is required for basic cellular homeostasis in silkworm cells.

Initial assessments in the BmE and BmN cell lines indicated that the phenotype of *SUGP1* deficiency was reproducible across different cell lineages, without lineage-specific artifacts. Although RT-qPCR assays showed varying degrees of transcript depletion, this is most likely due to the differential nonsense-mediated mRNA decay (NMD) responses existing among different cell lineages (Figure S1B). Given that BmE cells have higher editing efficiency and greater stability (Figure S1D), and their embryonic origin background can provide broader physiological relevance, we subsequently prioritized BmE cells for omics analysis.

Deep RNA-seq on *SUGP1*-KO BmE polyclonal cell populations revealed a broad impact on pre-mRNA splicing compared with WT BmE cells. We identified 105 significant AS events (rMATS FDR < 0.05; Figure 1C; Supplementary Table S2). These events spanned all five major classes of alternative splicing: skipped exons (SE), alternative 5′ and 3′ splice sites (A5SS/A3SS), retained introns (RI), and mutually exclusive exons (MXE) (Figure 1E). Untreated wild-type (WT) cells served as the primary baseline in all phenotypic and transcriptomic comparisons.

To rule out potential off-target effects of the sgRNA and false positives stemming from vector transfection, we included an empty vector transfection group (P200) as an auxiliary technical control and tested an independent secondary sgRNA. The secondary sgRNA (sgRNA4, CCCGGTCCAATGGGTCCTGG, targeting exon 3; editing efficiency ~13%) reproduced the splicing defects. Crucially, global transcriptome analysis showed that the pattern of alternative splicing dysregulation obtained when comparing *SUGP1*-KO BmE polyclonal cell populations with the empty vector control group (P200 vs. *SUGP1*-KO) was similar to the pattern observed with the wild type as the baseline (WT vs. *SUGP1*-KO) (Figure S2A,B). Moreover, the proportional breakdown across all five major alternative splicing classes remained remarkably consistent between the two comparisons (Figure S2C,D), confirming that the splicing defects were specifically attributable to *SUGP1* disruption rather than vector transfection artifacts.

We next asked whether *SUGP1*’s splicing regulatory function has a similar pattern in *Drosophila*. We crossed the *Drosophila* RNAi strain (VDRC: V25779) with a ubiquitous Gal4 driver (Tsinghua University: 00033) to achieve systemic *SUGP1* knockdown (SUGP1-KD), collecting synchronized 3-day-old adult flies (pooled at 5 males and 5 females per replicate) for transcriptomic profiling. *SUGP1* expression was significantly reduced in the knockdown flies (*p* = 0.0046; Figure S1C).

At the whole-organism level, *SUGP1* knockdown targeting exon 2 (resulting in an estimated ~70% reduction in *SUGP1* transcript levels; Figure S1C) also triggered extensive splicing defects (363 significant AS events; Figure 1D,F; Supplementary Table S2). Despite species-specific differences in the distribution of alternative splicing types, extensive splicing disruption occurred in both experimental systems, supporting a consistent effect of SUGP1 in maintaining pre-mRNA splicing fidelity in the two tested insect models.

### 3.2. SUGP1 Deficiency Alters Global Alternative Splicing and Transcriptomic Profiles

**Figure 2:**
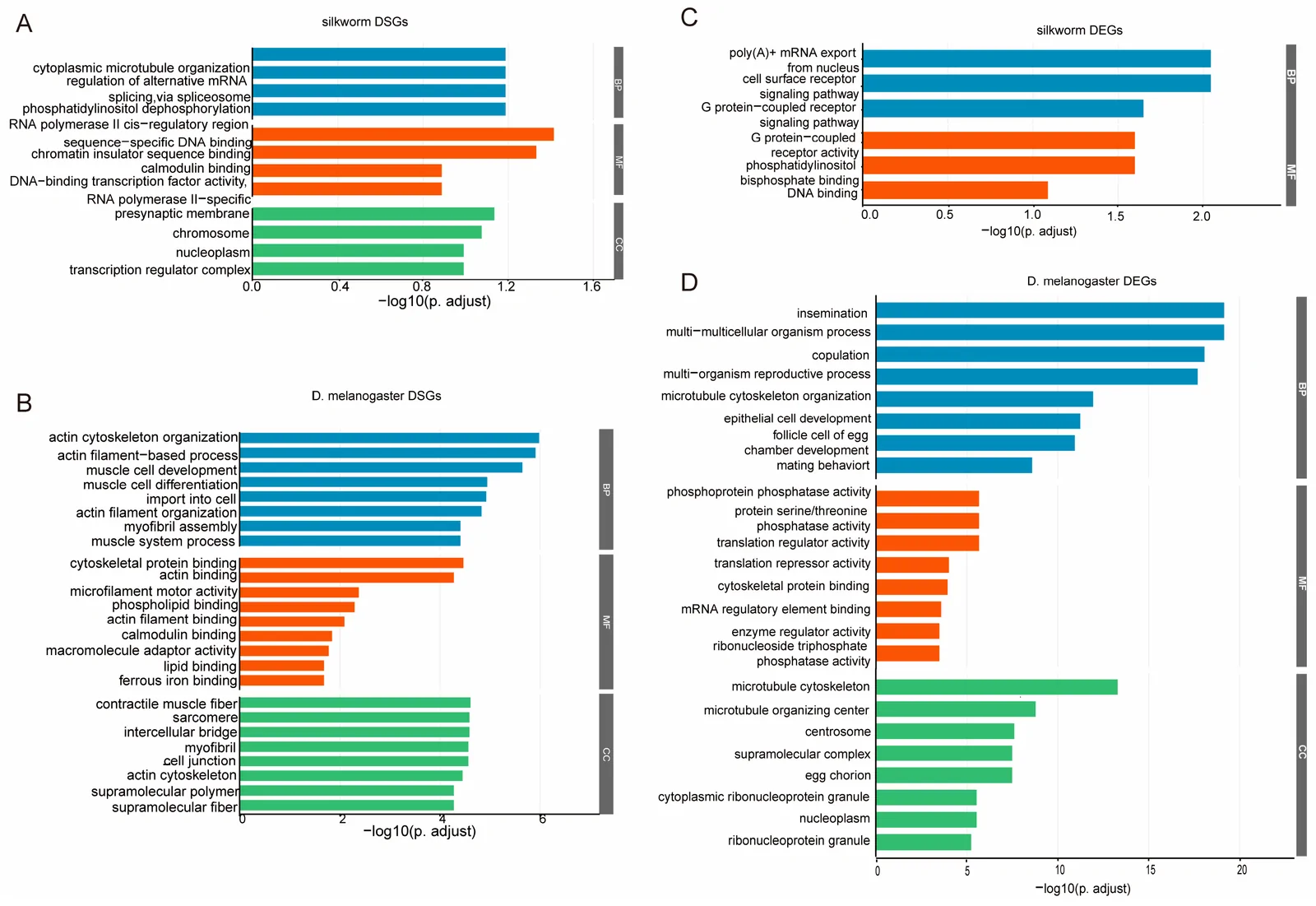
*SUGP1* deficiency alters global alternative splicing and transcriptomic profiles. (**A**,**B**) GO analysis of differentially spliced genes (DSGs) in polyclonal *B. mori BmE* cell populations (**A**) and pooled 3-day-old adult *D. melanogaster* (equal sex ratio, 5 males and 5 females; (**B**)). (**C**,**D**) GO analysis of differentially expressed genes (DEGs; vs. WT control) in polyclonal *B. mori* BmE cell populations (**C**) and pooled 3-day-old adult *D. melanogaster* (**D**). The −log10(*p*.adjust) is plotted on the *x*-axis with the top-ranked GO terms on the *Y*-axis. Bars are color-coded by functional category (BP, MF, and CC), with bar lengths reflecting statistical significance −log_10_(*p*.adjust).

After determining that *SUGP1* deficiency causes extensive splicing dysregulation, we next investigated which functional gene networks were most affected in each species. In *B. mori*, functional categorization of differentially spliced genes (DSGs) showed trends toward transcriptional and regulatory processes, with top-ranked GO categories including RNA polymerase II cis-regulatory region binding, chromatin insulator sequence binding, and alternative mRNA splicing regulation, although these GO terms did not reach statistical significance after multiple-testing correction (Figure 2A; Supplementary Table S3). In contrast, KEGG pathway analysis highlighted the circadian rhythm pathway (*p*.adjust < 0.05; Figure S3A; Supplementary Table S3), although this classification is supported by a very limited gene count. These analyses suggest that while broad functional classifications reflect regulatory associations, no specific pathway was robustly associated with *SUGP1*-dependent splicing in *B. mori*, which contrasts with the broader pathway-level perturbations observed in *D. melanogaster*. At the expression level, *SUGP1*-KO led to differential expression of 224 genes (Supplementary Table S3). GO analysis showed terms including poly(A)+ mRNA nuclear export and GPCR signaling (Figure 2C), whereas KEGG pathway analysis pointed toward the mRNA surveillance pathway and nucleocytoplasmic transport among the top-ranked categories (Figure S3C; e.g., involving *NXT1*), though the gene ratio for these terms is low. However, these categories are driven by isolated single genes rather than broad multi-gene coordinate regulation, and they did not exhibit robust statistical enrichment. Overall, these findings indicate that in *B. mori*, neither alternative splicing nor gene expression changes converge onto a specific, robustly enriched functional pathway. This contrasts notably with the broader, tissue-specific pathway-level perturbations observed in *D. melanogaster*, suggesting divergent regulatory architectures between the two species.

In *D. melanogaster*, the identified pathways reflect the multi-tissue composition of the whole 3-day-old adult flies, representing an overall organismal response rather than isolated cell-autonomous defects. DSGs were mainly associated with cytoskeletal structures, as shown by GO terms such as actin cytoskeleton organization and myofibril assembly (Figure 2B; Supplementary Table S3) and the KEGG pathway cytoskeleton in muscle cells (Figure S3B; Supplementary Table S3). DEGs were associated with reproductive processes and developmental pathways (Figure 2D; Supplementary Table S3), which was consistent with the significant reduction in effective reproductive capacity in SUGP1-KD *D. melanogaster* (Figure S3D,E; Supplementary Table S3). These profiles correspond to the heterogeneous composition of the adult flies, where non-germline structural tissues and reproductive organs represent the predominant RNA pools.

### 3.3. SUGP1 Deficiency Reduces Transposon-Derived piRNA Abundance

Although preliminary screenings have suggested the potential involvement of splicing factors in small RNA regulation [11,12,13,14], a direct link between these factors and piRNA biogenesis has not been previously reported. Building on the established role of *SUGP1* in pre-mRNA splicing fidelity [4,5,6], we next examined its impact on the piRNA pathway.

We performed small RNA sequencing on the cell samples from *SUGP1*-KO silkworm BmE polyclonal cell populations (Figure S1B,D,E). After trimming a 3′ adapter from the sequencing data, the structural RNAs (tRNA, miRNA, rRNA, snoRNA, snRNA) were filtered out. Given that transposon-derived piRNAs represent the primary effectors of transposon silencing and constitute the vast majority (50–80%) of the total piRNA library [19,49], while exhibiting distinct 1U/10A nucleotide biases and characteristic 10-nt ping-pong overlaps, we focused on these transposon-derived sequences. To identify authentic piRNAs and exclude other small RNAs or degradation fragments, only non-structural reads of 24–35 nucleotides that mapped to transposable elements were retained and designated as transposable element-derived piRNA-like small RNAs.

**Figure 3:**
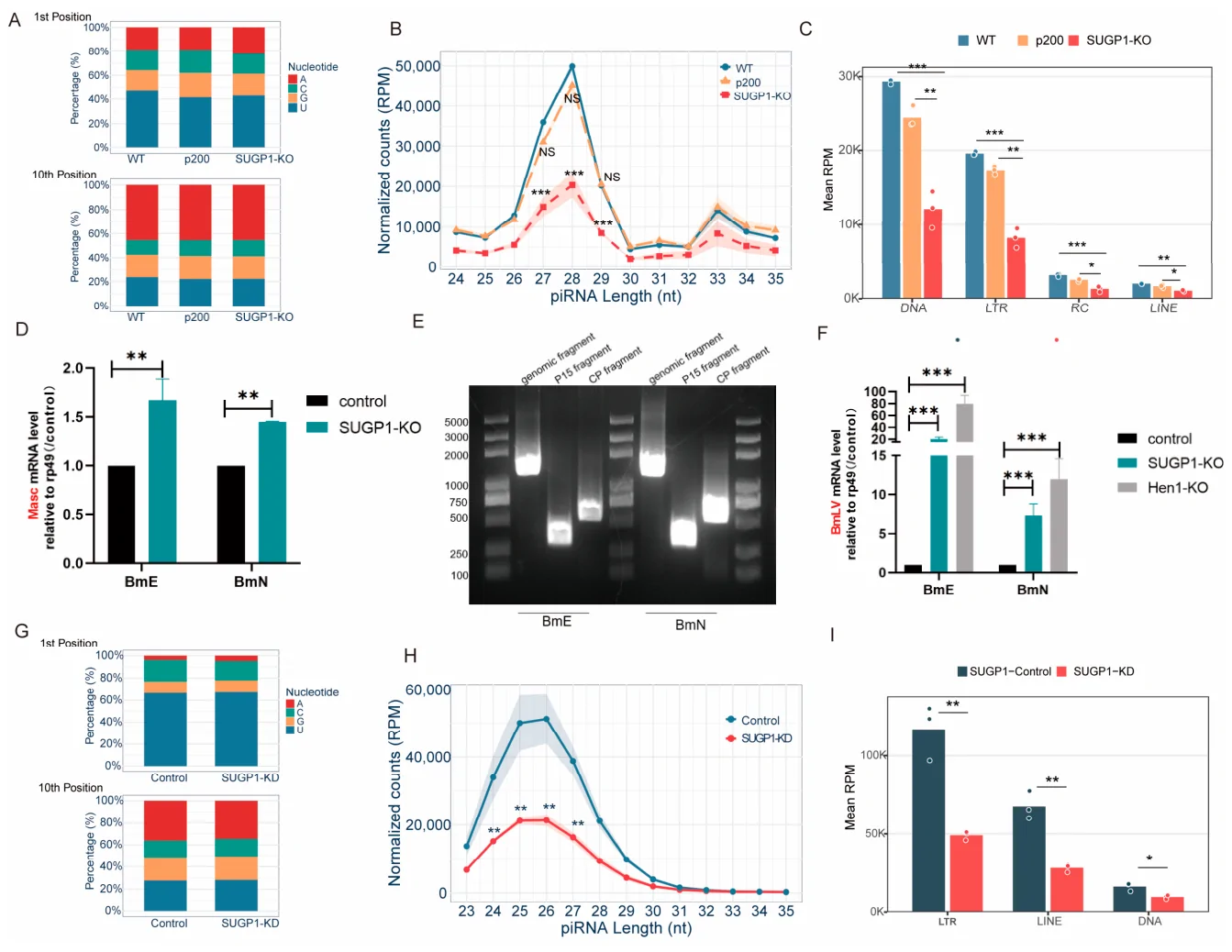
Functional Analysis of SUGP1 in piRNA Profile Maintenance and Host Immune Response. (**A**) Distribution characteristics of the first and 10th base of piRNA derived from transposons following SUGP1 perturbation in polyclonal *B. mori* BmE cell populations. Different color blocks represent the proportion of each base. (**B**) Overall analysis of the changes in transposon-derived piRNAs after SUGP1 perturbation: downregulation of transposon-derived piRNAs in polyclonal *B. mori* BmE cell populations. Shading represents biological replicate variation. (**C**) The profile depicts transposon types associated with reduced piRNAs following SUGP1 perturbation in polyclonal *B. mori* BmE cell populations. The *X*-axis displays transposon types, while the *Y*-axis represents the number of reduced piRNAs mapping to each transposon type. Bar colors correspond to control and experimental groups. Overlaid data points on bars indicate the variation range across biological replicates. (**D**) RT-qPCR quantification of *Masc*-RNA following *SUGP1*-KO in polyclonal *B. mori* BmE and BmN cell populations (sgRNA 1: TCTTGTACTCAAAGCAGATT targeting exon 1). (**E**) PCR detection of *BmLV* in BmE and BmN cells. Amplified regions include genes encoding the CP and P15 proteins, and an additional genomic segment. (**F**) Relative *BmLV* expression levels following *SUGP1*-KO polyclonal *B. mori* BmE and BmN cell populations (sgRNA 1: TCTTGTACTCAAAGCAGATT targeting exon 1) and *Hen1*-KO polyclonal *B. mori* BmE and BmN cell populations (sgRNA 1: CTAACGCGGTTAAAAGAGAC targeting exon 1), quantified by RT-qPCR assays using primers targeting the viral methyltransferase domain. Statistical significance was determined using a two-tailed Student’s *t*-test for (**D**) and Welch’s one-way ANOVA with Dunnett’s T3 post hoc test for (**F**). Data are presented as mean ± SD (derived from three independent biological replicates (*n* = 3)). (**G**) Distribution characteristics of the first and 10th base of piRNA derived from transposons following SUGP1 perturbation in pooled 3-day-old adult *D. melanogaster* (5 males and 5 females per replicate; SUGP1-KD). (**H**) Overall analysis of the changes in transposon-derived piRNAs after SUGP1-KD: downregulation of transposon-derived piRNAs in pooled adult *D. melanogaster*. Grey shading represents biological replicate variation. (**I**) The profile depicts transposon types associated with reduced piRNAs following SUGP1-KD in pooled adult *D. melanogaster*. In panels (**B**,**C**), significant symbols denote comparison of *SUGP1*-KO or empty vector (P200) with WT, and the significant symbols in (**H**,**I**) denote comparisons of SUGP1-KD with the RNAi control. Multiple unpaired two-tailed *t*-tests with Welch’s correction (without assuming equal variances) were used to determine the statistical differences in the small RNA length distribution profiles (**B**,**H**) and transposable element (TE) category abundances (**C**,**I**) across three biological replicates (*n* = 3). Multiple testing was controlled using the two-stage linear step-up false discovery rate (FDR) procedure of Benjamini, Krieger, and Yekutieli (BKY method). NS, not significant; * *p* < 0.05; ** *p* < 0.01; *** *p* < 0.001. Data are presented as mean ± SD.

The results show that these small RNAs displayed nucleotide preferences of piRNA molecules (a strong bias for uridine at the 1st nucleotide [1U] and adenosine at the 10th nucleotide [10A]) and the signature 10-nt 5′ overlap indicative of ping-pong amplification [32,51] (Figure 3A and Figure S4A). Following the normalization method described in the literature [49], the filtered total non-structural reads were used as the normalization factor to calculate piRNA abundance (RPM). Upon validation of piRNA identity, a reduction in transposon-derived piRNAs was observed in *SUGP1*-deficient BmE cells, primarily concentrated within the canonical piRNA size distribution of 26–30 nt (Figure 3B). Further analysis revealed that reduced piRNAs predominantly mapped to Class II transposons (DNA transposons) in *SUGP1*-deficient BmE cells, with varying degrees of mapping observed across other transposon categories (Figure 3C and Figure S4B,C). The quantitative downregulation of transposon-derived piRNAs provides the main observed molecular change associated with *SUGP1* deficiency in BmE cells.

To assess whether this molecular defect translates into downstream functional physiological consequences, we next examined the readouts of key pathways in SUGP1-deficient silkworm cells. We first examined a classic ping-pong cycle pair in *B. mori*, *Fem*-piRNA and *Masc*-RNA [32,52]. Quantitative assays (RT-qPCR assays) demonstrated that *Masc*-RNA levels were significantly upregulated in *SUGP1*-KO polyclonal BmE and BmN cell populations (Figure 3D), which is consistent with compromised piRNA-mediated target repression, although this is an indirect functional readout. Furthermore, the viral load of the endogenous silkworm virus *BmLV* (*Bombyx mori* latent virus) serves as a sensitive indicator for the integrity of the siRNA and piRNA antiviral surveillance pathway [51]. We detected the *BmLV* genomic sequence in silkworm cells and confirmed that knocking out the known piRNA pathway gene *Hen1* (sgRNA1: CTAACGCGGTTAAAAGAGAC, targeting exon 1; estimated editing efficiency approximately 27%; Figure S4D,E) led to a dramatic escalation in *BmLV* load (Figure 3E,F). Notably, a significant increase in *BmLV* viral load was also observed following *SUGP1* deficiency; although the magnitude of this upregulation was less pronounced than that in the *Hen1*-KO group, it remained statistically significant and highly consistent (Figure 3F). Although these indirect assays cannot provide direct mechanistic evidence for piRNA biogenesis defects, the coordinated upregulation of *Masc*-RNA and *BmLV* abundance consistently reflects the downstream functional consequences associated with impaired piRNA surveillance.

Meanwhile, to investigate whether *SUGP1* deficiency elicits a similar phenotype of piRNA dysregulation in *D. melanogaster*, we performed small RNA-seq on pooled 3-day-old adult *D. melanogaster* in which gene silencing at the RNA level was caused by systemic *SUGP1*-KD. Small RNA analysis was carried out according to the above-mentioned procedure: structural RNAs were filtered out, and reads of 23–35 nt mapping to transposable elements (TEs) were retained for downstream analysis. Consistent with the findings in *B. mori*, they exhibited typical piRNA characteristics, especially a strong preference for 1U and 10A (Figure 3G and Figure S4A). Consistent with the overall trend observed in the silkworm cell populations, systemic SUGP1-KD in the whole *Drosophila* body led to a significant reduction in transposon-derived piRNAs (Figure 3H) (in *Drosophila*, the length of piRNA usually peaks at 25–26 nt). Reduced piRNAs predominantly mapped to LTR (Figure 3I and Figure S4F,G). Although the analysis of the whole animal captures the combined responses of somatic and germ cell compartments rather than isolated cell-autonomous readouts, these consistent reductions indicate that the link between *SUGP1* functional perturbation and decreased piRNA abundance is biologically reproducible across different insect models.

### 3.4. Identification of SUGP1-Interacting Proteins Implicates Its Roles in RNA Processing and Translation

**Figure 4:**
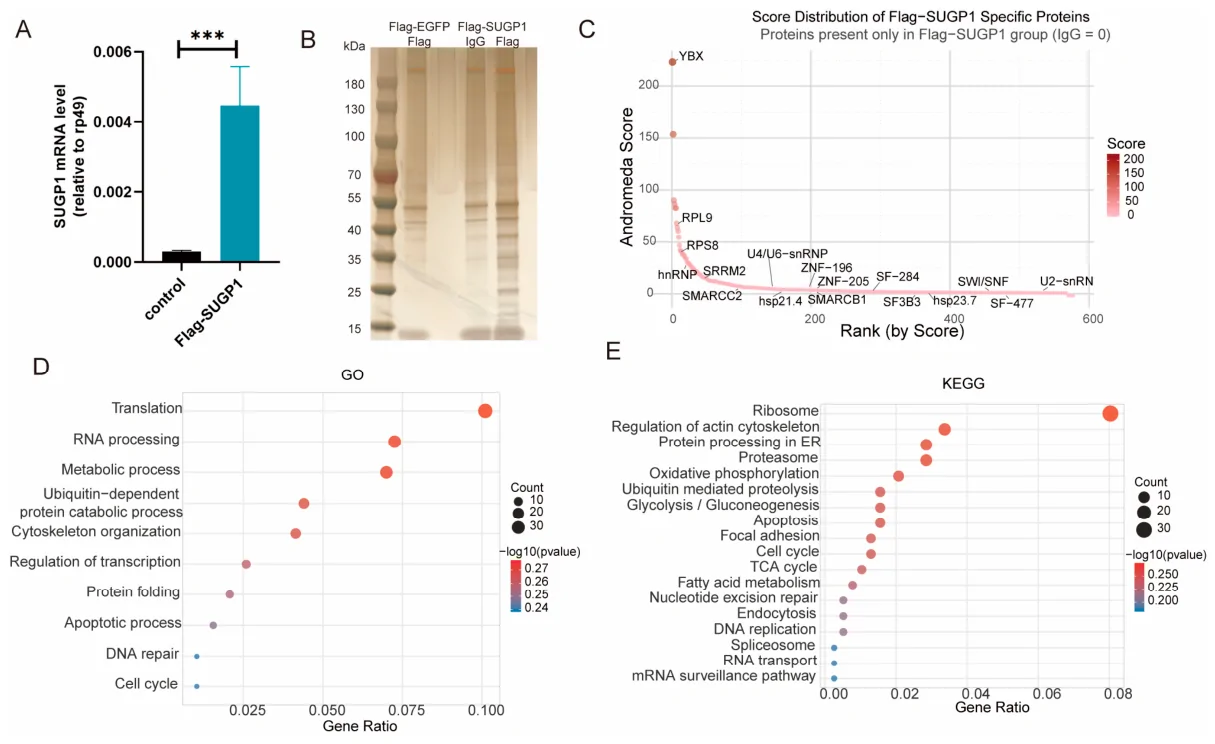
Identification of SUGP1-interacting proteins implicates its roles in RNA processing and translation. (**A**) RT-qPCR assays show that Flag-SUGP1 is significantly expressed after transfection into BmE cells. The wild-type (WT) was used as the control. Statistical analysis: two-tailed Student’s *t*-test. Data are presented as mean ± SD (derived from three independent biological replicates (*n* = 3)). (**B**) Silver staining results of the immunoprecipitation complexes from *B. mori* BmE cell lysates. Compared with the parallel negative controls of Flag-EGFP and IgG, obvious protein products were present in the Flag-SUGP1 eluate (sample). Proteins that could be identified in the Flag-SUGP1 immunoprecipitation product and were completely absent in the negative control were defined as candidate interaction partners of SUGP1. (**C**) Ranked distribution of candidate SUGP1-interacting proteins identified by IP-MS. The *Y*-axis represents the Andromeda protein score (a peptide-spectrum matching confidence score generated by the MaxQuant pipeline; see Methods for details). The *X*-axis displays the ranked order of these corresponding proteins based on their Andromeda scores. (**D**) GO analysis of proteins identified in the SUGP1 IP-MS experiment. (**E**) KEGG analysis of proteins identified in the SUGP1 IP-MS experiment. In both (**D**,**E**), the *X*-axis represents the enrichment ratio, while the *Y*-axis displays the identified GO/KEGG terms. Bubble size corresponds to the number of proteins annotated for each term. A gradient color scale indicates the statistical significance (*p*-value), with deeper red hues denoting higher degrees of significance (*** *p* < 0.001).

**Figure 5:**
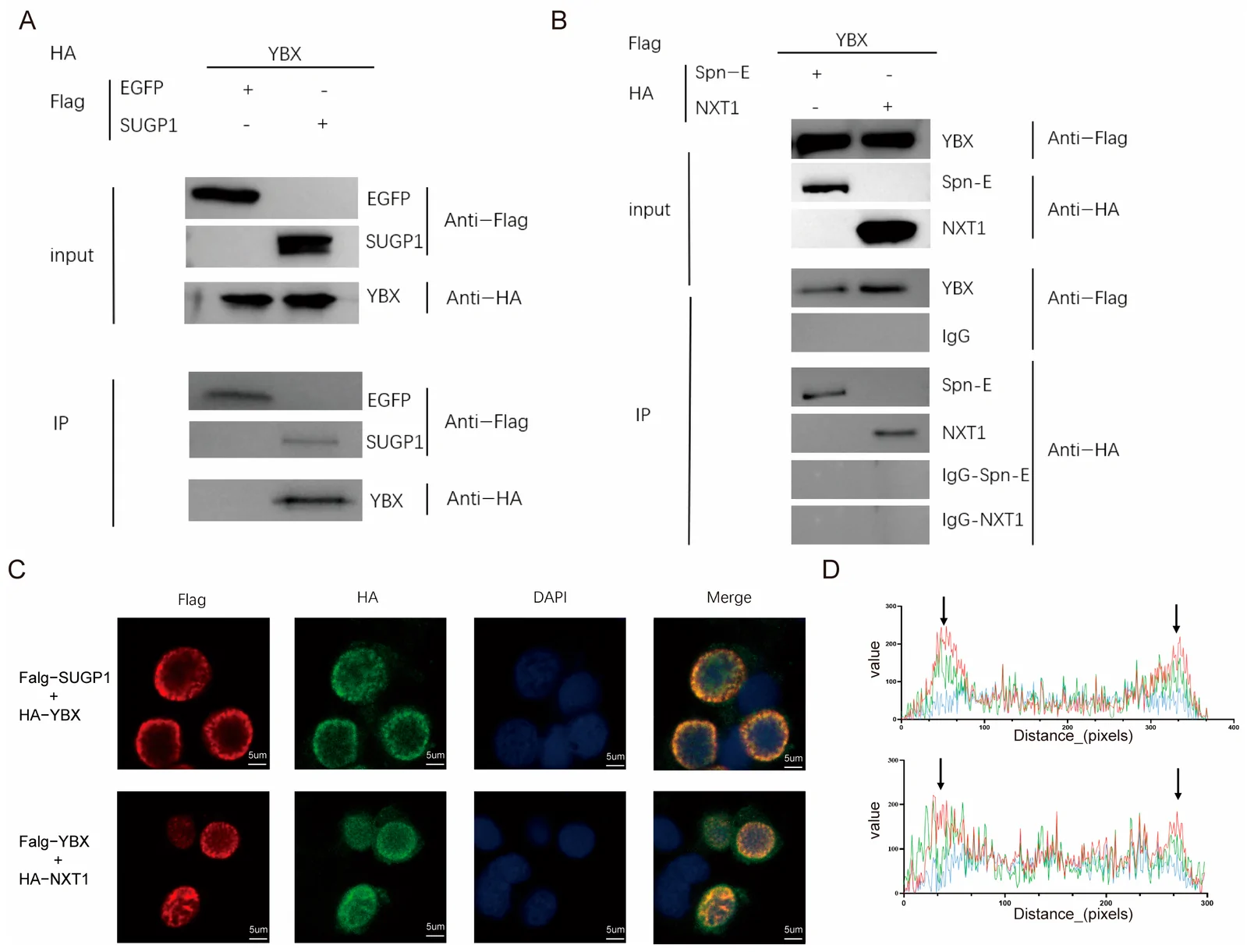
Physical and functional interactions between SUGP1 and the multifunctional RBP YBX. (**A**) Co-immunoprecipitation (Co-IP) and Western blot (WB) assay confirming the interaction between SUGP1 and YBX in *B. mori* BmE cells. Expression vectors with Flag and HA tags were co-transfected into the cells. Whole cell lysates (Input) were evaluated to confirm protein expression, followed by immunoprecipitation using anti-Flag magnetic beads (IP: Flag; Flag-EGFP as a negative control). Immunoprecipitated complexes were detected with anti-HA and anti-Flag antibodies (IB: HA, Flag). Expected product sizes: Flag-SUGP1 (~115 kDa), Flag-EGFP (~28 kDa), and HA-YBX (~40 kDa). The figure shows one representative image of three independent biological replicates (*n* = 3). (**B**) Co-IP and WB analysis indicated a physical association between YBX and NXT1 and Spn-E. WB analysis (IB: HA, Flag) was performed on whole cell lysates (Input) and anti-Flag immunoprecipitates (IP: Flag; mouse IgG as a negative control) of co-transfected BmE cells. Expected product sizes: Flag-YBX (~40 kDa), HA-Spn-E (~150 kDa), and HA-NXT1 (~17 kDa). Representative blots from three independent biological replicates (*n* = 3) are shown. (**C**) Immunofluorescence shows the co-localization of SUGP1 and YBX, YBX and NXT1. Scale bar represents 5 μm. (**D**) Quantification of fluorescence intensity for colocalization: ImageJ was used to quantify fluorescence intensity of colocalized regions in (**C**). Line-scan measurements along the central axis showed that fluorescence signal was significantly enriched within the overlapping region. The arrows indicate the fluorescence intensity at the overlapping positions.

To identify the molecular partners mediating the cellular functions of SUGP1, we performed co-immunoprecipitation combined with mass spectrometry (Co-IP/MS) in *B. mori* BmE cells, leveraging their established advantages in piRNA research [15,23,38,40]. A Flag-tagged SUGP1 vector was transfected into BmE cells (Figure S5A). Both RT-qPCR assays (Figure 4A) and Western blotting (Figure 5A) confirmed the successful expression and specific enrichment of Flag-SUGP1. Silver staining further verified the specificity of enrichment relative to the Flag-EGFP and IgG controls (Figure 4B). Mass spectrometry-based identification of interacting proteins demonstrated high reproducibility across three independent biological replicates, and core interacting proteins were markedly enriched relative to the IgG negative control. In the IgG negative control, 376 non-specific background-binding proteins were detected. We retained the proteins specifically present in the Flag-SUGP1 eluate (absent in IgG) for analysis, and a total of 386 such interacting proteins were identified (Figure 4C; Supplementary Table S4). Gene Ontology (GO) analysis of these 386 proteins pointed to functional clusters such as RNA processing, translation, metabolic regulation, and ubiquitin-dependent protein catabolism (Figure 4D; Supplementary Table S5), although no GO term reached statistical significance after multiple-testing correction. At the molecular function level, these proteins were mainly involved in RNA binding, protein binding, and DNA binding (Figure S5B). KEGG pathway analysis categorized the identified interacting proteins across multiple functional groups, including the ribosome, spliceosome, and RNA transport pathways (Figure 4E; Supplementary Table S5). Among the 386 identified interacting proteins, the Y-box protein (YBX) yielded the highest Andromeda score (Figure 4C), making it a key candidate protein for subsequent mechanistic studies.

### 3.5. Physical and Functional Interactions Between SUGP1 and the Multifunctional RBP YBX

The physical interaction between SUGP1 and YBX was confirmed by Co-IP combined with Western blotting (Figure 5A). Given that YBX is a typical multifunctional RBP with known roles in transcriptional regulation, mRNA stability, and translational control [41,42,43,44], we further investigated its broader interaction profile with factors involved in RNA transport and downstream processing. Co-IP assays confirmed the physical association between YBX and the mRNA export factor NXT1 (Figure 5B). In addition, given that YBX coordinates stress granule (SG) assembly [53], we also investigated its association with Spn-E, a known piRNA pathway component. Co-IP similarly confirmed the physical interaction between YBX and Spn-E (Figure 5B). While these pairwise Co-IP results show individual binary associations rather than the simultaneous assembly of a single multi-protein complex, they indicate that YBX possesses the biochemical capacity to interact with both mRNA export machinery and piRNA-associated factors.

To determine whether these physical associations are supported by spatial proximity in intact cells, we systematically examined their subcellular distribution using confocal immunofluorescence (Figure 5C,D and Figure S5D). Consistent with the Co-IP results, steady-state imaging revealed clear co-localization between SUGP1 and YBX, with Manders’ overlap coefficients across 34 individual cells yielding M1 = 0.8960 ± 0.0526 (fraction of HA-YBX signal overlapping with Flag-SUGP1) and M2 = 0.8507 ± 0.0656 (fraction of Flag-SUGP1 overlapping with HA-YBX) (Figure 5C,D). Spatial co-localization analysis likewise confirmed substantial overlap between YBX and NXT1 (M1 = 0.8760 ± 0.0669 for HA-NXT1 overlapping with Flag-YBX; M2 = 0.8235 ± 0.0702 for Flag-YBX overlapping with HA-NXT1; *n* = 30 cells) (Figure 5C,D and Figure S5D). Although optical co-localization at confocal resolution does not by itself establish direct molecular contact, the spatial proximity is consistent with the Co-IP results.

**Figure 6:**
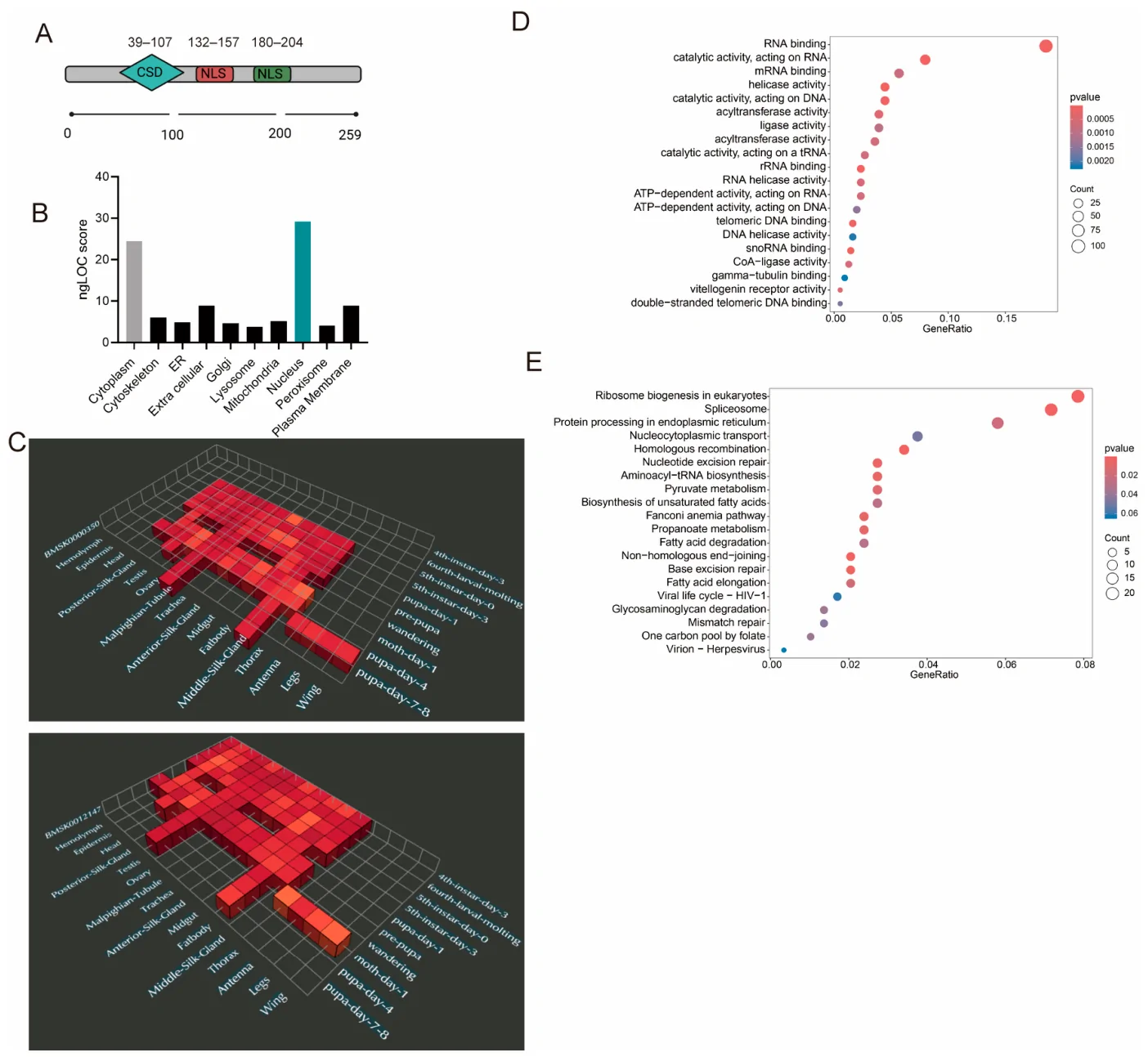
Bioinformatics Characterization and Transcriptomic Impact of YBX in Silkworm. (**A**) Layout of the predicted nuclear localization signal (NLS) sites and key domains, including the cold-shock domain (CSD), within the YBX sequence. (**B**) Subcellular Localization of YBX (Data sourced from SilkDB 3.0). The *X*-axis indicates the predicted subcellular localization category, and the *Y*-axis represents the score (Generated by the ngLOC algorithm, representing Posterior Probability/Weight) at the corresponding position. YBX is mainly localized in the cytoplasm and the nucleus. (**C**) Tissue and Temporal RNA expression profile of *YBX* (**top**) and *SUGP1* (**bottom**) (Data sourced from SilkDB 3.0). Heatmap displaying transcript abundance across diverse tissues (*X*-axis) and developmental stages (*Y*-axis), with red gradients reflecting elevated expression levels. (**D**) Biological Process in GO analysis of DEGs after *YBX*-KO. (**E**) KEGG bubble chart of DEGs following *YBX*-KO. In (**D**,**E**) The *X*-axis represents the enrichment ratio. The *Y*-axis shows GO and KEGG terms. Bubble size corresponds to the number of DEGs annotated to each GO and KEGG term, while color represents the *p*-value, with deeper red hues denoting smaller *p*-values.

In line with this subcellular distribution, predictive structural scores support the nucleocytoplasmic dual-localization potential of YBX (Figure 6A,B), reinforcing the view that SUGP1 and YBX share a compatible compartment at the nucleocytoplasmic interface. Moreover, in silkworms, *YBX* RNA is widely expressed in various tissues and developmental stages, similar to that of *SUGP1* (Figure 6C).

To assess whether the physical association between SUGP1 and YBX is consistent with biological processes, we constructed *YBX*-deficient polyclonal cell populations using CRISPR/Cas9-mediated gene editing targeting exon 3 (Figure S6A). Successful targeted disruption was confirmed by Sanger sequencing and subsequent ICE deconvolution analysis, with an estimated editing efficiency of approximately 23%, which mainly generated loss-of-function frameshift deletions (Figure S6B,C). Subsequently, RNA-seq was performed on these *YBX*-deficient cells and matched wild-type (WT) control cells using the same sequencing platform and analysis pipeline as the *SUGP1* deficiency study. Transcriptomic analysis identified a total of 464 differentially expressed genes (DEGs; |log2FC| ≥ 1, FDR < 0.05) upon *YBX* disruption. Cross-comparative analysis revealed an overlap of 12 DEGs between the *YBX*-deficient dataset and the SUGP1-deficient dataset (Supplementary Table S7).

Functional categorization analysis of the *YBX*-KO transcriptome and the SUGP1 IP-MS interactome showed common pathway characteristics, including RNA binding/processing, ATP-dependent helicase activity, ribosome biogenesis, spliceosome components, protein processing in the ER, and nucleocytoplasmic transport (Figure 6D,E; Supplementary Table S6). Although these parallel pathway-level categorizations do not provide direct genetic evidence of functional redundancy in the absence of epistasis analysis or rescue experiments, the coordinated performance among these categories is consistent with SUGP1 and YBX operating within an interconnected RNA-processing and ribonucleoprotein network.

### 3.6. piRNA Reduction as a Proposed Secondary Consequence of SUGP1-KO–Associated Stress

**Figure 7:**
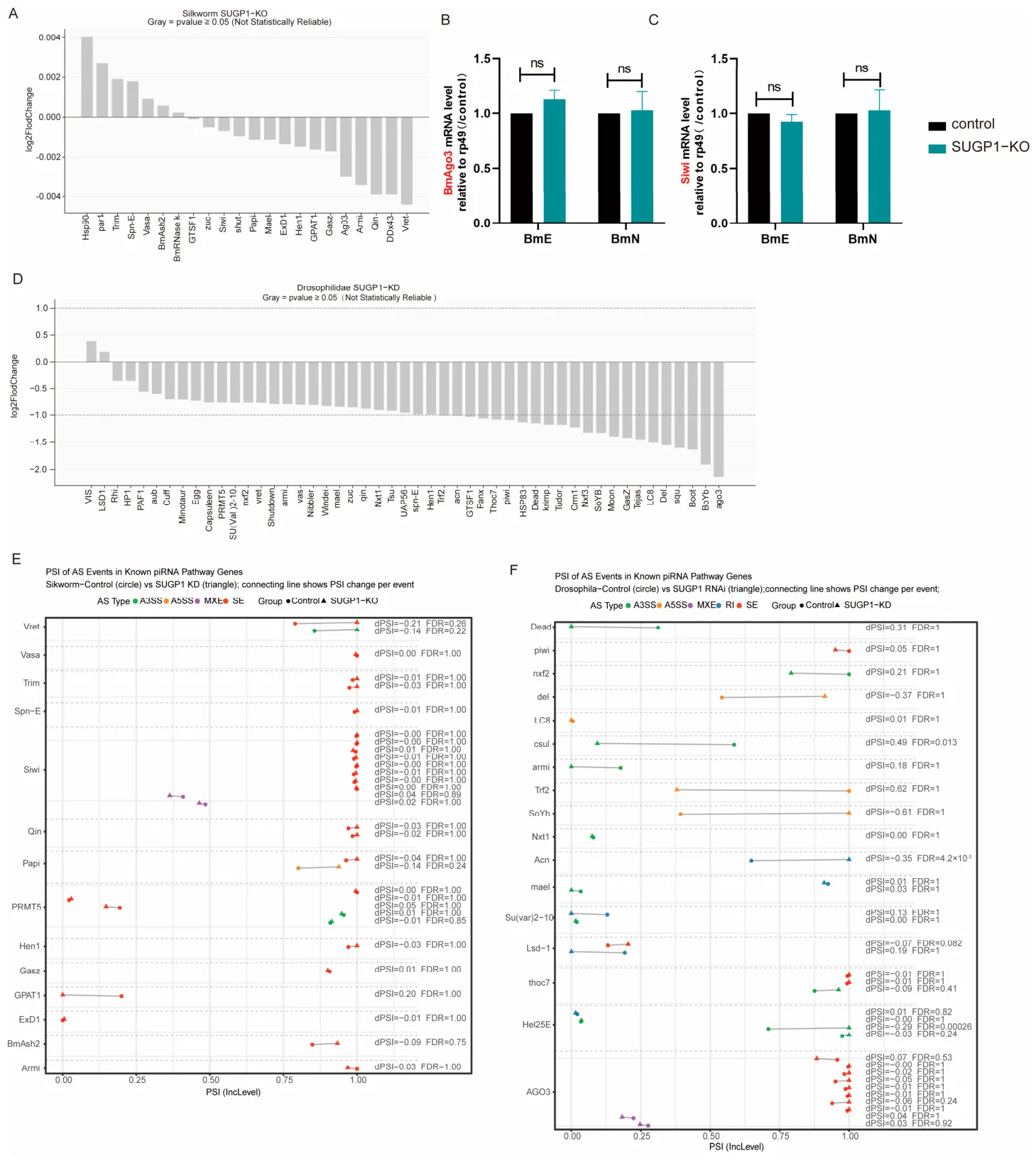
Expression and splicing profiles of known piRNA pathway genes after SUGP1 dysregulation. (**A**) RNA-seq analysis of *B. mori SUGP1*-KO samples to examine the expression changes of the known components in the piRNA pathway. All |log2FoldChange| values are less than 1. (**B**,**C**) RT-qPCR assay results indicate changes in *Siwi*/*BmAgo3* expression levels following *SUGP1*-KO. For all panels, expression levels are normalized to the *Bmrp49* reference gene. Data are presented as mean ± SD (*n* = 3 independent replicates). Statistical significance was determined using a two-tailed Student’s *t*-test (ns, not significant, where applicable; no comparisons reached statistical significance). (**D**) RNA-seq quantification of known piRNA pathway components in *D. melanogaster SUGP1*-KD samples. (**E**,**F**) Alternative splicing profiles of known piRNA biogenesis pathway genes following *SUGP1*-KO in *B. mori* (**E**) and SUGP1-KD in *D. melanogaster* (**F**). The lines connecting the control and treatment points indicate the shift in PSI (Percent Spliced In, quantifying the relative inclusion ratio of alternative exons).

*SUGP1*-KO reduces piRNA abundance, but the question of direct versus indirect mechanism remains unclear. In this study, we systematically integrated *B. mori* RNA-seq (transcriptome) and Flag-SUGP1 IP-MS (interactome) data to evaluate potential regulatory links across four dimensions: (1) At the expression level, differential gene expression analysis showed no significant fluctuations in the expression of known piRNA pathway genes in silkworms (e.g., *Siwi*, *BmAgo3*) and RT-qPCR confirmed this (Figure 7A–C and Figure S7A,B), indicating that *SUGP1*-KO does not discernibly perturb the transcriptional levels of known piRNA pathway genes. (2) rMATS analysis showed that most piRNA biogenesis factors did not produce aberrant splice isoforms (Figure 1C and Figure S2A,B). Most of the ΔPSI values directly showed negligible changes with FDR values far from significant (Figure 7E,F and Figure S7C,D), suggesting that *SUGP1* may not affect piRNA biogenesis by regulating the splicing of known pathway components. (3) At the protein interaction level, IP-MS under our affinity-purification conditions did not recover known piRNA pathway proteins among SUGP1-associated factors (Supplementary Table S4). (4) In pathway analysis, GO and KEGG analysis of both RNA-seq and IP-MS data failed to highlight any significant enrichment of piRNA-related terms, such as “RNA silencing” or the “RISC complex” (Figure 2A,C, Figure S3A, Figure 4D,E and Figure S5B,C; Supplementary Table S3 and Table S5). None of these four lines produced positive evidence for a direct SUGP1–piRNA biogenesis link. This pattern fits an indirect model, though we cannot exclude transient or context-dependent interactions below the detection threshold.

To provide a conceptual framework for future research, we propose a hypothetical working model. In this model, the reduction in piRNAs in *SUGP1-KO* polyclonal cell populations may correlate with systemic cellular perturbations through three speculative and non-mutually exclusive possibilities. First, *SUGP1-KO*-induced splicing dysregulation is associated with a global cellular stress response; this is supported by changes in mRNA surveillance and protein quality control systems in the RNA-seq data (Figure 2C and Figure S3C; Supplementary Table S3), and IP-MS revealed SUGP1 associations with heat shock proteins (Hsp21.4, Hsp23.7), the proteasome, and stress granule (SG)-associated proteins (Figure 4C; Supplementary Table S4). Notably, the mRNA levels of known piRNA factors (Siwi, BmAgo3) were unchanged (Figure 7A–C) despite reduced piRNA output. This observation raises the possibility that post-transcriptional constraints might be involved, although direct measurement of translation efficiency still remains to be verified. The involvement of SG regulator YBX [53], together with the observation that “ribosome” is categorized as a pathway in both the SUGP1 IP-MS (Figure 4E) and *YBX*-KO transcriptome (Figure 6E), provides correlative hints that stress-associated translational reprogramming could be a contributing factor. Second, we hypothesize that alterations in RNA export pathways might indirectly influence piRNA biogenesis. While Co-IP validated binary interactions between YBX and the export factor NXT1 (Figure 5B–D and Figure S5D), and ‘RNA transport’ was identified as a category across both omics datasets (Figure 4E and Figure 6E), whether nucleocytoplasmic export of piRNA precursors or pathway transcripts is functionally compromised in vivo warrants direct experimental verification. Third, chromatin-level perturbations represent another speculative possibility: SUGP1’s physical associations with SWI/SNF and PBAF complexes (Figure 4C) and the categorization of Pol II-associated transcription factor networks among the DSGs (Figure 2A) raise the possibility that chromatin accessibility and transcriptional activity at piRNA cluster loci might be perturbed, a premise that requires future analysis of piRNA cluster chromatin states.

## 4. Discussion

### 4.1. SUGP1 as a Consistent Splicing Regulator with Implications for piRNA Homeostasis

Most prior work on SUGP1 has been carried out in mammalian cells, particularly in the context of *SF3B1* mutations in cancer, where SUGP1 links SF3B1 to the RNA helicase DHX15 during splicing quality control [4,5,6]. Our data show that *SUGP1* deficiency leads to extensive abnormal splicing in *B. mori* and *D. melanogaster*, confirming its role in maintaining transcript isoform balance (Figure 1C,D). These two species exhibit different alternative splicing (AS) patterns. We attribute this divergence mainly to the differences in biological systems—a homogeneous, rapidly dividing embryonic cell line versus highly differentiated whole-adult tissues—rather than fundamental differences in the mechanism of SUGP1 function. The silkworm cell line captures an immediate, quality-control-dominated response to splicing damage, mRNA surveillance and nucleocytoplasmic transport pathways were identified among the top-ranked DEG-associated categories (Figure 2C and Figure S3C), though the gene counts supporting these terms are limited. The observations in adult *Drosophila* integrate long-term, tissue-level effects that focus on structurally critical and tissue-abundant transcripts (cytoskeleton and reproductive genes; Figure 2B,D and Figure S3B,D,E). By comparing the DEGs and DSGs between SUGP1-dysregulated silkworm and *Drosophila*, we identified a subset of significantly altered orthologs in both species (Supplementary Table S7). These orthologous targets offer a useful starting point for understanding *SUGP1*’s dual role in splicing regulation and piRNA abundance. Despite the differences in alternative splicing patterns and downstream transcriptomic features, both models show a consistent down-regulation of transposon-derived piRNAs (Figure 3A–I), suggesting that *SUGP1*, as a splicing regulator, may indirectly influence broader RNA metabolism and subsequently impair piRNA homeostasis.

### 4.2. SUGP1-Dependent RNA Processing Is Linked to piRNA Abundance

Large-scale genetic screens in *Drosophila* and *C. elegans* have repeatedly turned up spliceosome components as genes whose deletion leads to transposon derepression [11,12,13,14]. Whether these factors act directly within small RNA pathways or simply sustain the RNA-processing environment that piRNA production depends on remains incompletely understood. In our data, *SUGP1* fits the latter architecture: splice-site surveillance is functionally connected to the maintenance of piRNA abundance.

We probed *SUGP1*’s connection to piRNA biogenesis through four independent lines of evidence—expression, splicing, physical interaction, and pathway analysis—and none produced a positive signal linking *SUGP1* to the known piRNA biogenesis machinery (Results, Section 3.6). *SUGP1-KO* reshaped the splicing landscape (Figure 1C,D), but the ΔPSI values for *Siwi* and *BmAgo3* hovered near zero, with FDR values far above significance (Figure 7E,F and Figure S7C,D). Their transcriptional levels were likewise unaffected (Figure 7A–D and Figure S7A,B). This result implies that although global splicing is widely disrupted, piRNA pathway transcripts are hardly affected, raising the possibility that post-transcriptional or indirect regulatory constraints may be involved rather than quantitative transcript depletion. The *Hen1*-KO control provides an internal calibration: *Hen1* is a piRNA biogenesis factor, and its deletion leads to a significant increase in the *BmLV* load in silkworms compared with the increase observed in *SUGP1-KO* polyclonal cell populations (Figure 3F). This quantitative difference is consistent with an indirect or auxiliary role for SUGP1 rather than direct core biogenesis activity. Although we cannot completely rule out the existence of transient or low-abundance interactions below the detection sensitivity of IP-MS, considering all the results, the effect is more likely to represent an indirect downstream systemic perturbation.

Reduced cell viability and marked vacuolization observed in *SUGP1-KO* polyclonal cell populations (Figure 1A,B) prompted us to consider whether the reduction in piRNA is specifically due to *SUGP1* deficiency or secondary to general cellular stress. Although empty vector transfection caused a slight decrease in viability, *SUGP1*-KO induced a further significant reduction, confirming that the disruption of SUGP1 impairs cell fitness. The stable mRNA levels of core pathway genes (*Siwi*, *BmAgo3*; Figure 7A–C) ruled out the possibility of non-specific global degradation caused by dying cells. The normalization strategy of small RNA libraries based on RPM reflected the selective depletion of piRNAs derived from transposable elements (TE) rather than a bulk yield artifact. However, given that *SUGP1* deficiency leads to extensive splicing abnormalities and is associated with stress-related genes (Figure 1C and Figure 4C), *SUGP1* deficiency may indirectly impair piRNA homeostasis through these intermediate factors. Our findings on the specific splicing-related factor SUGP1 provide an alternative perspective for interpreting previous screening data: some positive hits related to RNA processing identified in large-scale screens may intersect with specialized small RNA pathways through secondary, systemic alterations in basal RNA metabolism rather than direct pathway participation.

### 4.3. The SUGP1 and YBX Physical Association Implicates a Splicing–RNA Export–Translation Surveillance Module

Among the 386 SUGP1-interacting proteins identified by IP-MS, YBX was the top-scoring hit (Figure 4C). YBX is a well-characterized multifunctional RNA-binding protein [41,42,43,44]. Its dual localization in the nucleus and cytoplasm (Figure 6A,B) and its widespread expression in various tissues and developmental stages of silkworms (Figure 6C) are consistent with its roles in coordinating general RNA metabolism. Therefore, the physical interaction between SUGP1 and YBX verified by Co-IP and co-localization (Figure 5A,C,D) raises the possibility that YBX may serve as a potential partner of SUGP1 in RNA-related processes.

The transcriptome profile of *YBX*-KO provides additional context. Genes differentially expressed upon *YBX*-KO are categorized into functional groups including ribosome biogenesis, spliceosome function, protein processing, and nucleocytoplasmic transport (Figure 6D,E), similar to the categories identified in the IP-MS of SUGP1 data (Figure 4D,E). This functional convergence suggests that SUGP1 and YBX may participate in overlapping RNA metabolic pathways, rather than acting in completely unrelated biological processes. Furthermore, pairwise interactions of YBX with NXT1 and Spn-E (Figure 5B) demonstrate binary biochemical associations with RNA export and stress-associated factors, respectively. We regard these observations as relevant clues rather than conclusive evidence of a coordinated functional axis. The precise biological role of YBX in this context remains speculative, and the biochemical coordination between these interaction partners, and whether it is constitutive or stress-induced, remains to be addressed in future studies.

### 4.4. A Proposed Regulatory Model Linking SUGP1-Dependent RNA Processing to piRNA Homeostasis

Based on the above results, we propose the following hypothetical working model to provide a conceptual basis for future research. Under this speculative scenario, loss of *SUGP1* impairs splicing fidelity, which may correlate with cellular stress response characterized by the presence of mRNA surveillance, heat shock, and proteasome-related factors in our RNA-seq and IP-MS datasets (Figure 4C and Figure S3C). Under such stress-associated conditions, pairwise interactions between YBX and factors such as NXT1 (Figure 4E, Figure 5B and Figure 6E) raise the possibility that nucleocytoplasmic RNA distribution might be broadly perturbed, which could theoretically affect the availability of piRNA-related transcripts. Meanwhile, the physical association of SUGP1 with chromatin-remodeling complexes (SWI/SNF, PBAF; Figure 4C) and the categorization of RNA polymerase II-associated transcription factor networks among the top-ranked DSG GO terms (Figure 2A) lead to the speculative possibility that chromatin accessibility or transcription at piRNA cluster loci might be affected. In this framework, the decrease in piRNA levels is hypothesized to reflect multi-level disruptions in basal RNA metabolic homeostasis rather than the loss of a canonical pathway component. Importantly, we emphasize that this working model remains strictly hypothetical. The proposed links (potential translational bottlenecks, alterations in RNA distribution, or perturbations at the chromatin level) represent possibilities inferred from correlative omics maps. Direct and specific functional validation is still required to clearly establish these proposed mechanisms.

### 4.5. Limitations and Future Perspectives

The mechanistic conclusions of this study are subject to several important limitations. First, the proposed cascade model is based on the correlation between transcriptomics and interactomics; the causal relationships between individual nodes have not been verified. Performing ATAC-seq analysis on the chromatin accessibility of piRNA clusters in *SUGP1*-KO polyclonal cell populations would enable independent evaluation of chromatin-level perturbations. In addition, measuring the translation efficiency of mRNAs in the known piRNA pathway based on ribosome profiling analysis would enable independent evaluation of translational bottlenecks. Second, our silkworm knockout experiments utilized polyclonal edited cell populations rather than single-cell clones, which inherently retain some degree of cellular heterogeneity. Third, due to the lack of commercially available, high-quality *B. mori*-specific SUGP1 antibodies, it is not possible to directly verify the knockout of *SUGP1* at the protein level. Therefore, our verification of the knockout relies on Sanger sequencing, RT-qPCR assays, RNA-seq read alignments, and phenotypic evidence (Figure S1 and Figure 1A,B). Future work will use epitope-tagged endogenous knock-in lines or mass spectrometry quantification targeting *SUGP1* peptides to address this shortcoming. Fourth, biological differences exist between the experimental systems used (cultured silkworm embryo cells and whole-body samples of adult Drosophila), which likely contribute to divergence in their alternative splicing patterns.

## 5. Conclusions

Taken together, our data suggest the SUGP1 and YBX association is functionally linked to transcriptomic stability and co-transcriptional splicing in insects, and that its perturbation leads to systemic dysregulation associated with altered piRNA abundance. Integrated multi-omics analysis of the transcriptome and interactome suggests that *SUGP1* acts as a key regulator of global RNA processing homeostasis. Under this proposed model, loss of SUGP1 disrupts pre-mRNA splicing and cellular RNA-processing programs, thereby weakening the RNA regulatory environment needed to maintain piRNA abundance. These findings support a relationship between *SUGP1*-dependent RNA processing and piRNA abundance, with the detailed mechanism remaining to be validated, and underscore the challenge of separating core biogenesis factors from upstream RNA-processing regulators in complex RNA networks.

## Data Availability

Sequencing datasets have been deposited in the NCBI Sequence Read Archive (SRA) under BioProject accession PRJNA1337577.

## Supplementary Materials

The following supporting information can be downloaded at: https://www.mdpi.com/article/10.3390/biology15201852/s1, Figure S1: Validation of SUGP1 genetic manipulation efficiency in *B. mori* polyclonal cell populations and *D. melanogaster* strains; Figure S2: The alternative-splicing changes following SUGP1 dysregulation are not attributable to sgRNA-specific off-target effects or to the empty vector; Figure S3: The resulting landscapes of differential splicing and expression; Figure S4: Validation of Hen1 knockout in *B. mori* and normalized subcategory analysis of transposon-derived piRNA changes in *B. mori* and *D. melanogaster*; Figure S5: Vector construction, functional analysis, and subcellular localization of SUGP1 and YBX; Figure S6: Validation of YBX genetic manipulation efficiency in *B. mori* cells; Figure S7: Expression and splicing profiles of known piRNA pathway genes after SUGP1 dysregulation with an independent sgRNA; Table S1: SUGP1_Primer; Table S2: Alternative_splicing_summary_by_event_type; Table S3: Functional_analysis_of_DEGs_and_DSGs; Table S4: SUGP1_IP_MS_identified_proteins; Table S5: Functional_analysis_of_SUGP1_interactors; Table S6: Functional_analysis_of_YBX_KO_DEGs; Table S7: Ortholog_analysis_and_DEG_overlap; Supplementary File S1: Quality_metrics_and_data_deposition; Supplementary File S2: Uncropped_original_western_blots; Supplementary File S3: Uncropped_original_gel_images.
