# Supplementary material for: SUGP1 Deficiency Leads to Splicing Dysregulation and Reduced piRNA Abundance in Insects
Source: Biology (Basel). 2026 Oct 15;15(20):1852. doi: 10.3390/biology15201852 (PMC13701588; doi:10.3390/biology15201852)
Supplement: Supplementary file 1 [file biology-15-01852-s001.zip › Supplementary figures.pdf]

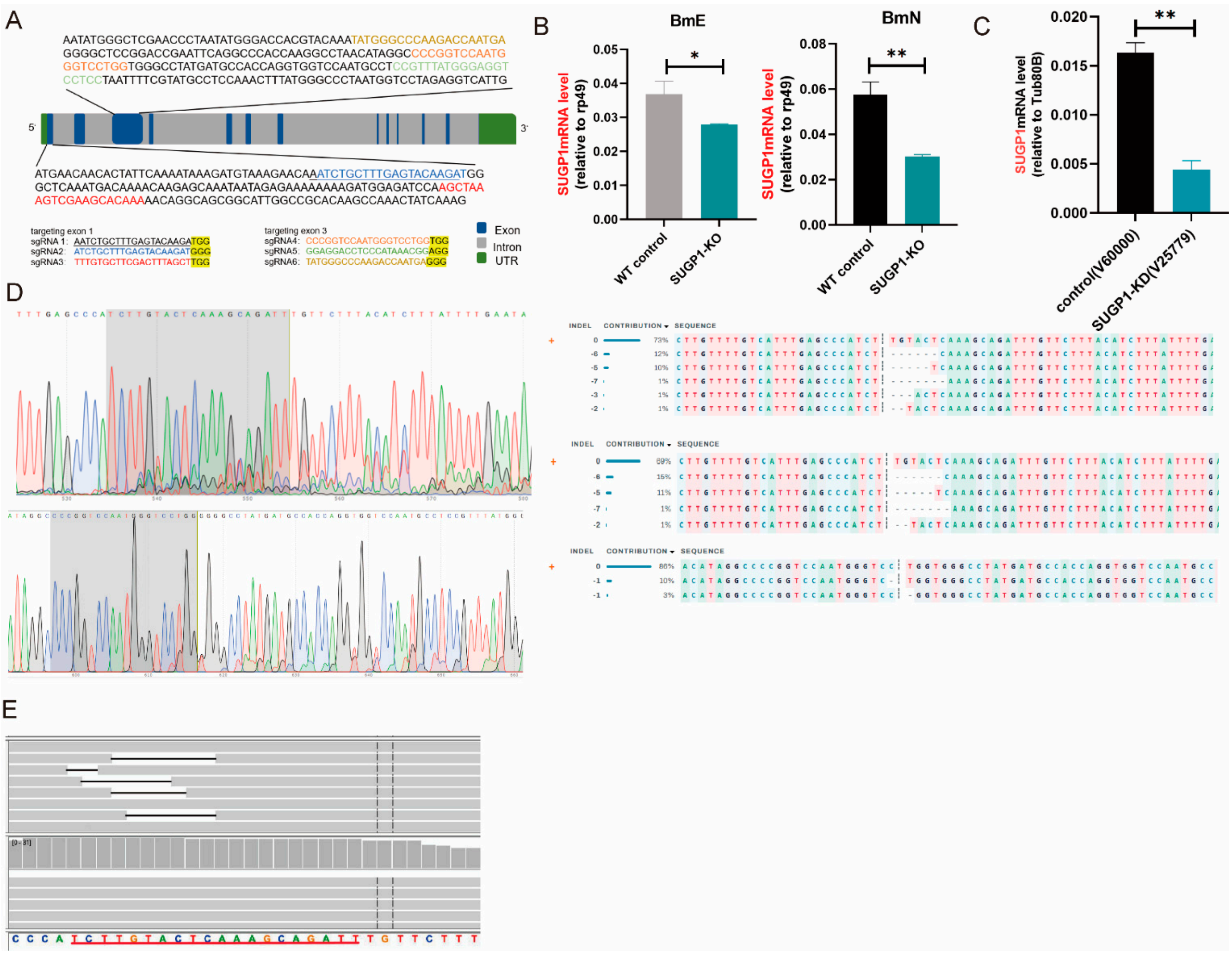

Figure S1. Validation of SUGP1 genetic manipulation efficiency in *B. mori* polyclonal cell populations and *D. melanogaster* strains.

(A) Gene structure of SUGP1 and the design of sgRNA targeting sites. Exons, introns, and untranslated regions (UTRs) are represented by blue, gray, and green boxes/lines, respectively. The sequences of exons 1 and 3 are shown in the figure, in which the three specific sgRNAs targeting exon 1 are highlighted with blue text, red text, and underline, respectively, and the three sgRNAs targeting exon 3 are indicated with orange, green, and yellowish-brown text, respectively. The detailed sequence of sgRNA (the first 20 bases) and its corresponding protospacer adjacent motif (PAM, highlighted in yellow) are provided below the genomic sequence. The three on the left target exon 1, and the three on the right target exon 3. (B) RT-qPCR analysis of SUGP1 mRNA levels in

control and CRISPR/Cas9-mediated SUGP1-KO silkworm BmE and BmN polyclonal cell populations. The Y-axis indicates the relative expression levels of *SUGP1* normalized to the internal reference gene *rp49*. The wild-type (WT) was used as the control group. (C) RT-qPCR validation of SUGP1-KD efficiency in adult *D. melanogaster* whole-body samples (dsRNA-GD10294; targeting exon 2 at 3R: 5,488,737–5,489,053 within the SUGP1 locus [3R: 5,485,543–5,489,489, + strand]). The data of the control group are from the V60000 strain (the standard genetic control strain of V25779). In these assays, Bmrp49 and Tub80B were utilized as the internal references for normalization in silkworms and *D. melanogaster*, respectively. ns: No significant difference; *P < 0.05; **P < 0.01; ***P < 0.001. Data are shown as the mean ± Standard Deviation (SD) derived from three independent replicates. (D) Representative Sanger sequencing chromatogram of sgRNA target sites within the SUGP1 locus (sgRNA1: TCTTGTACTCAAAGCAGATT targeting exon 1; sgRNA 4: CCCGGTCCAATGGGTCCTGG targeting exon 3). The gray shading indicates the location of the sgRNA target. The presence of distinct overlapping peaks downstream of the cleavage site demonstrates successful targeted genomic disruption and Indel formation. The editing efficiency can be obtained by reading the Sanger sequencing results through an online deconvolution tool (https://ice.editco.bio/). On the right is the representative result of Sanger sequencing chromatogram reading: BmN cells targeted by sgRNA 1 (top panel), and BmE cells targeted by sgRNA 1 and sgRNA 4 across independent biological tests (panels 2–3). Target-site editing efficiencies ranged between 13% and 28% across edited silkworm polyclonal cell populations, predominantly yielding frameshift deletions (primarily –6, –5, and –1 bp) from three independent biological experiments (n = 3). (E) Integrative Genomics Viewer (IGV) tracks displaying a representative diagram of the genomic editing status at the target site in the RNA-Seq

results following silkworm SUGP1 deficiency (n = 3 independent experiments). The alignment reads spanning the sgRNA1 (TCTTGTACTCAAAGCAGATT targeting exon 1) region (indicated by the red line) exhibit a significant base deletion gap, characterized by a disrupted region connected by a black horizontal line. 48 sequencing reads were detected, among which 29 contained indels and 6 contained base substitutions. Five representative reads are shown in the figure. The target site is on chromosome 21: 7,061,564 - 7,061,623.

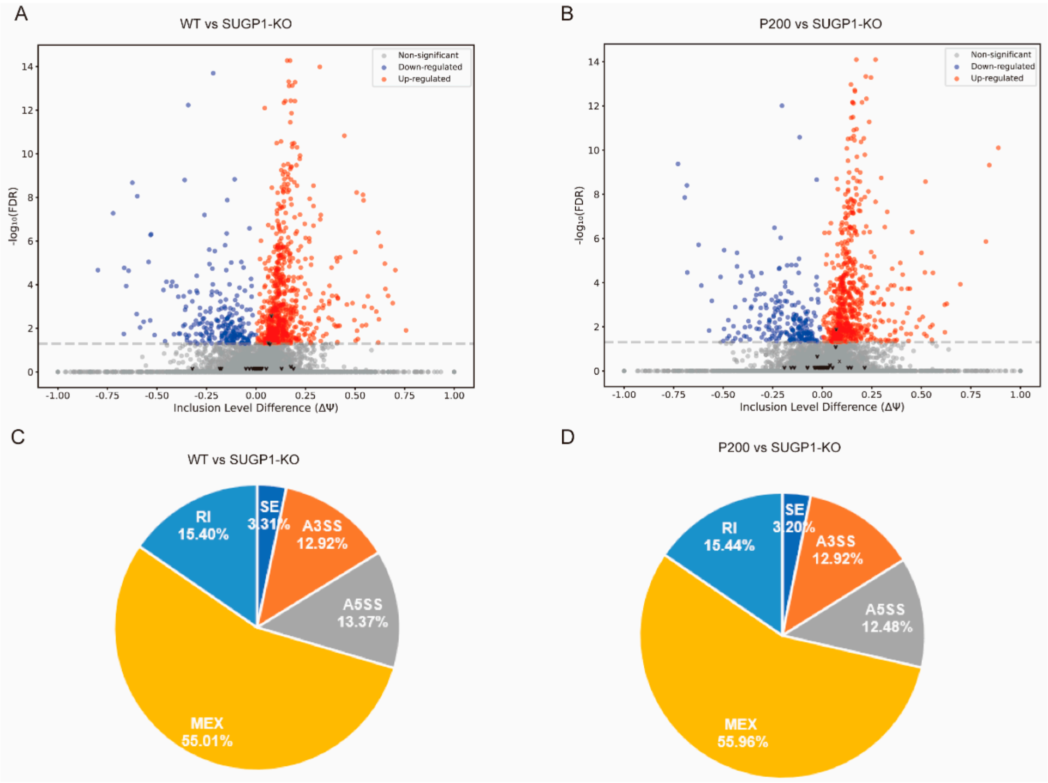

Figure S2. The alternative-splicing changes following SUGP1 dysregulation are not attributable to sgRNA-specific off-target effects or to the empty vector.

(A-B) Alternative splicing events after knockout with an independent sgRNA targeting the third exon of *B. mori* SUGP1. The X-axis shows the difference in splicing efficiency between SUGP1-KO and control groups, and the Y-axis represents -log10(FDR). Red and blue dots indicate significantly altered splicing events. Known genes in the piRNA biogenesis pathway are indicated

by black arrows. The control is WT in (A) and empty vector (P200) in (B). (C-D) Proportion of major classes of alternative splicing events: exon skipping (SE), alternative 5' splice site (A5SS), alternative 3' splice site (A3SS), retained intron(RI), and mutually exclusive exons (MXE). The control is WT in (C) and empty vector (P200) in (D).

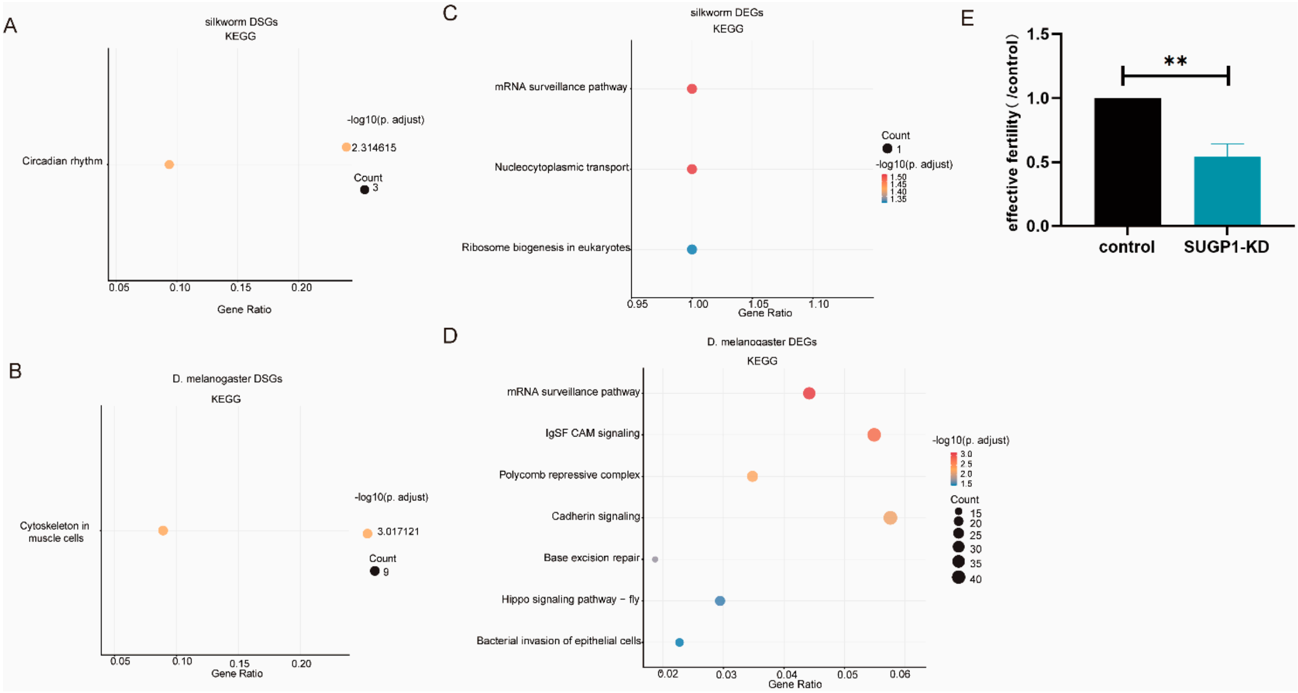

Figure S3. The resulting landscapes of differential splicing and expression.

(A-B) KEGG analysis of differentially spliced genes (DSGs) in *B. mori* (A) and *D. melanogaster* (B). The color gradient represents the statistical significance (p.adjust), where deeper red hues denote a higher degree of significance. (C-D) KEGG analysis of differentially expressed genes (DEGs) in *B. mori* (C) and *D. melanogaster* (D). The x-axis represents the enrichment ratio, while the y-axis denotes the identified KEGG terms. The bubble size corresponds to the number of differentially expressed genes annotated to each term. (E) Effective reproductive capacity of *Drosophila* following SUGP1-KD (V25779). The capacity is defined as the proportion of oviposited

eggs that successfully develop into viable adults. Values are normalized to the control (V60000). Data are presented as mean ± SD (n = 3 independent replicates). Statistical significance was determined using a two-tailed Student's t-test (ns, not significant; *P < 0.05, **P < 0.01, ***P < 0.001).

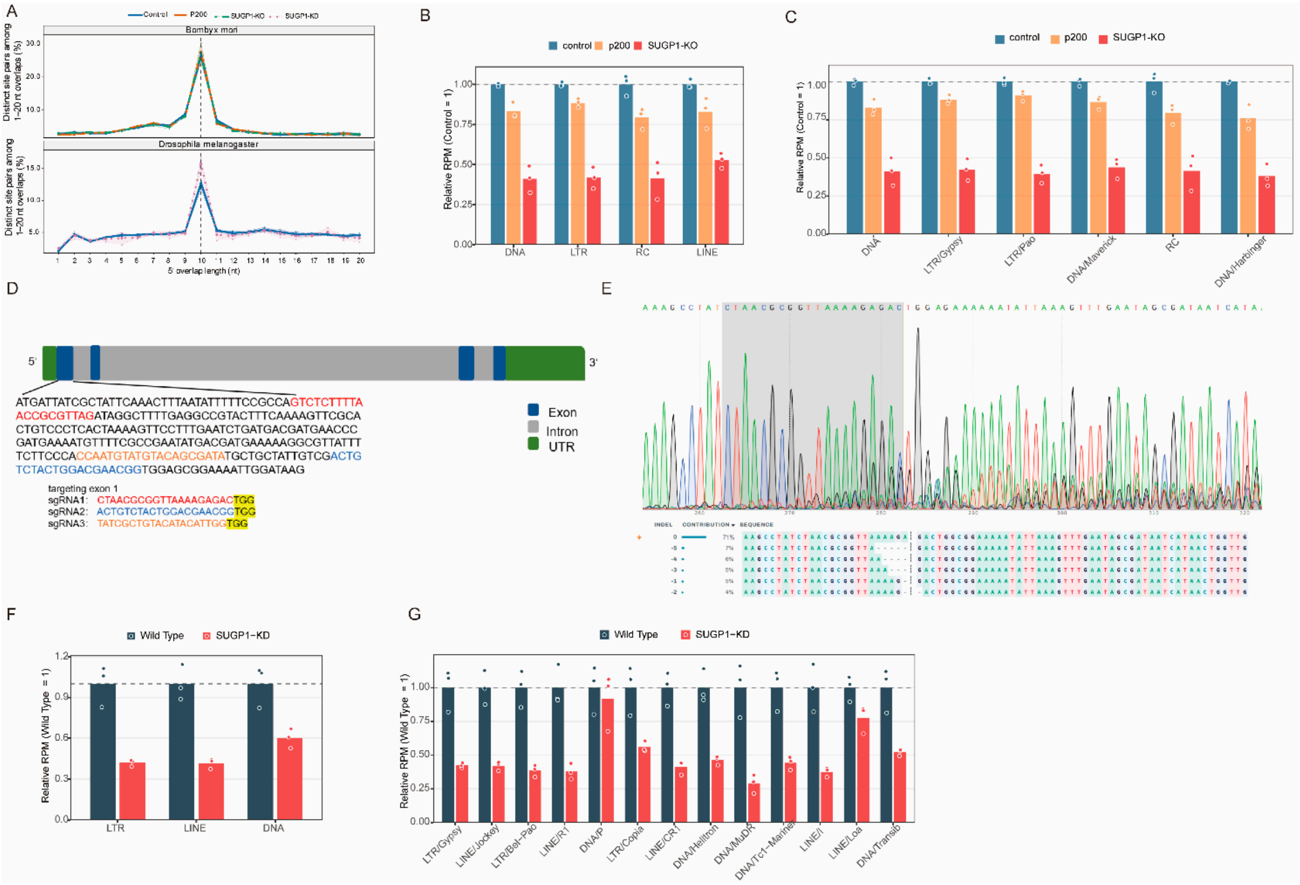

Figure S4. **Validation of Hen1 knockout in *B. mori* and** **normalized subcategory analysis of transposon-derived piRNA changes in *B. mori* and *D. melanogaster*.**

(A) Distribution of 5′ overlap lengths (1–20 nt) between opposite-strand, TE-derived piRNA-like small RNAs pairs in *B. mori* (top panel) and *D. melanogaster* (bottom panels 2–3). The sharp, prominent peak at 10 nt (dashed vertical line) indicates the canonical ping-pong amplification signature. Thin lines represent individual biological replicates, thick lines indicate group means, and

shaded ribbons denote mean ± SD. (B) Data are normalized by setting the control group value to 1 for each transposon type, allowing direct visual comparison of SUGP1-KO-induced changes across transposon categories in *B. mori* (corresponding to Figure 3C). (C) Transposon types are further refined into subcategories relative to panel (B). (D) Gene structure of *B. mori* Hen1 and the design of sgRNA targeting sites. Exons, introns, and untranslated regions (UTRs) are represented by blue, gray, and green boxes, respectively. The sequence of exon 1 is shown in the figure, in which the three specific sgRNAs targeting exon 1 are highlighted with blue text, red text, and orange text, respectively. The detailed sequence of sgRNA (the first 20 bases) and its corresponding protospacer adjacent motif (PAM, highlighted in yellow) are provided below the genomic sequence. (E) Representative Sanger sequencing chromatogram of sgRNA target sites within the Hen1 locus of the silkworm (sgRNA1: CTAACGCGGTTAAAAGAGAC targeting exon 1). The gray shading indicates the location of the sgRNA target. The presence of distinct overlapping peaks downstream of the cleavage site demonstrates successful targeted genomic disruption. Bottom panel: the editing efficiency can be obtained by reading the Sanger sequencing results through an online deconvolution tool (https://ice.editco.bio/). Target-site editing efficiency was approximately 27%, predominantly yielding frameshift deletions (primarily –5, –4, –3, and –1 bp) from three independent biological experiments (n = 3). (F) Data are normalized by setting the control group (V60000 strain) value to 1 for each transposon type, allowing for direct visual comparison of SUGP1-KD-induced (V25779) changes across transposon categories in *D. melanogaster* (corresponding to Figure 3I). (G) Transposon types are further refined into subcategories relative to panel (F).

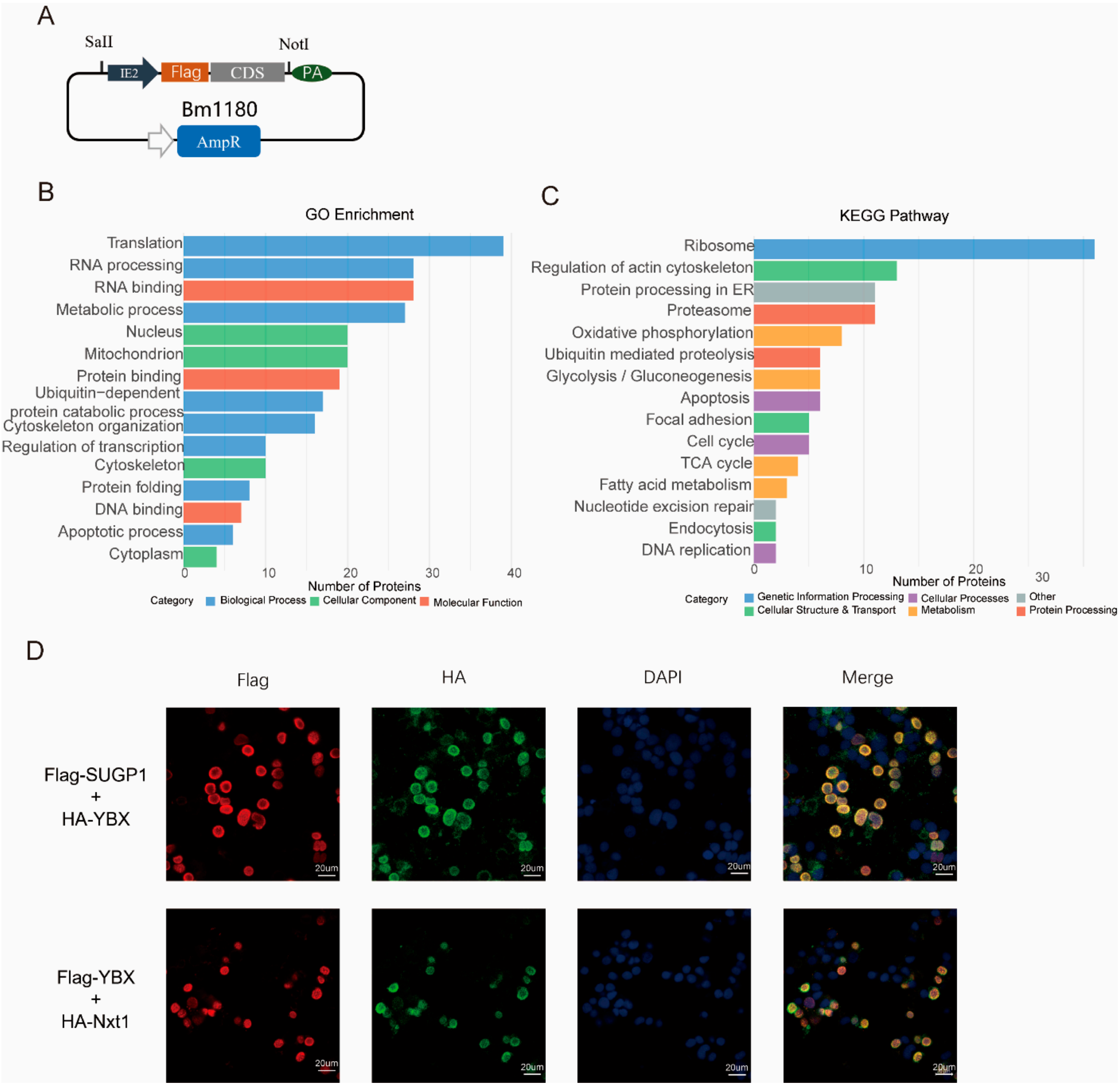

Figure S5. Vector construction, functional analysis, and subcellular localization of SUGP1 and YBX.

(A) Schematic diagram of vector construction. (B-C) For panels (B) and (C), corresponding to Figure 4D-E, a detailed categorical visualization of the identified GO/KEGG terms is provided. The Y-axis displays the GO/KEGG terms, while the X-axis indicates the number of proteins annotated for each term. Bars are color-coded according to their respective functional categories. (D) Immunofluorescence images demonstrating the co-localization of SUGP1 and YBX, and YBX and NXT1. Scale bar represents 20 μ m.

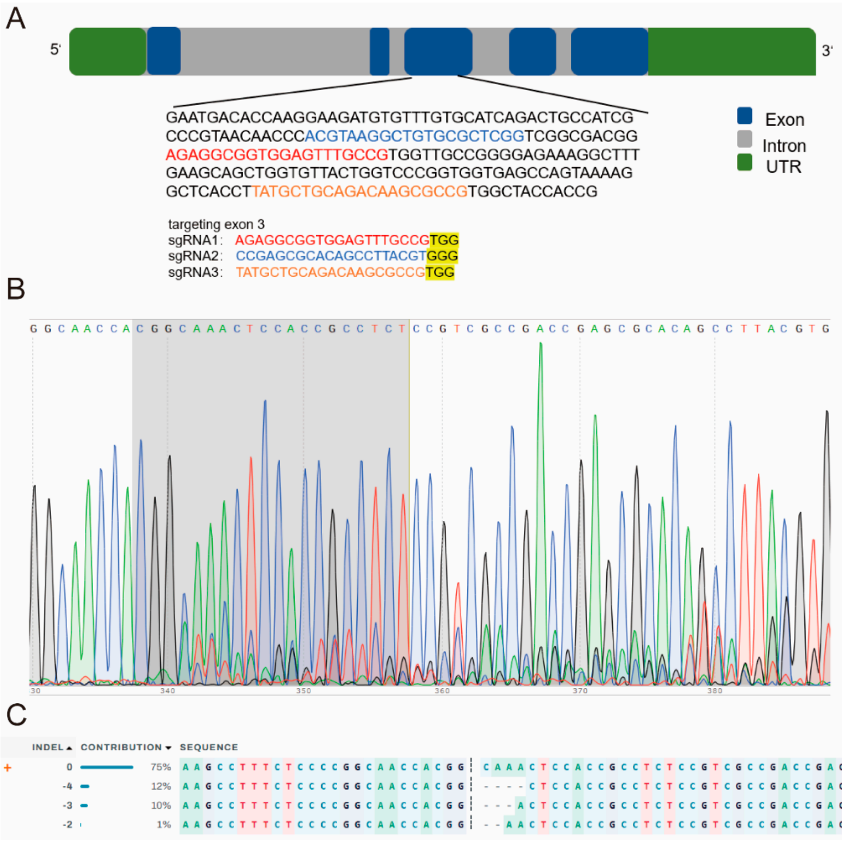

Figure S6. Validation of YBX genetic manipulation efficiency in *B. mori* cells.

(A) Gene structure of YBX and the design of sgRNA targeting sites. Exons, introns, and untranslated regions (UTRs) are represented by blue, gray, and green boxes/lines, respectively. The sequence of exon 3 is shown in the figure, in which the three specific sgRNAs targeting exon 3 are highlighted with blue text, red text, and orange text, respectively. The detailed sequence of sgRNA (the first 20 bases) and its corresponding protospacer adjacent motif (PAM, highlighted in yellow) are provided below the genomic sequence. (B) Representative Sanger sequencing chromatogram of sgRNA target sites within the YBX locus (sgRNA1: AGAGGCGGTGGAGTTTGCCG targeting exon 3). The gray shading indicates the location of the sgRNA target. The presence of distinct overlapping peaks downstream of the cleavage site demonstrates successful targeted genomic disruption and Indel formation. (C) The editing efficiency can be obtained by reading the Sanger sequencing results through an online deconvolution tool (https://ice.editco.bio/). Target-site editing efficiency was

approximately 23%, predominantly yielding frameshift deletions (primarily –4, –3, and –2 bp) from three independent biological experiments (n = 3).

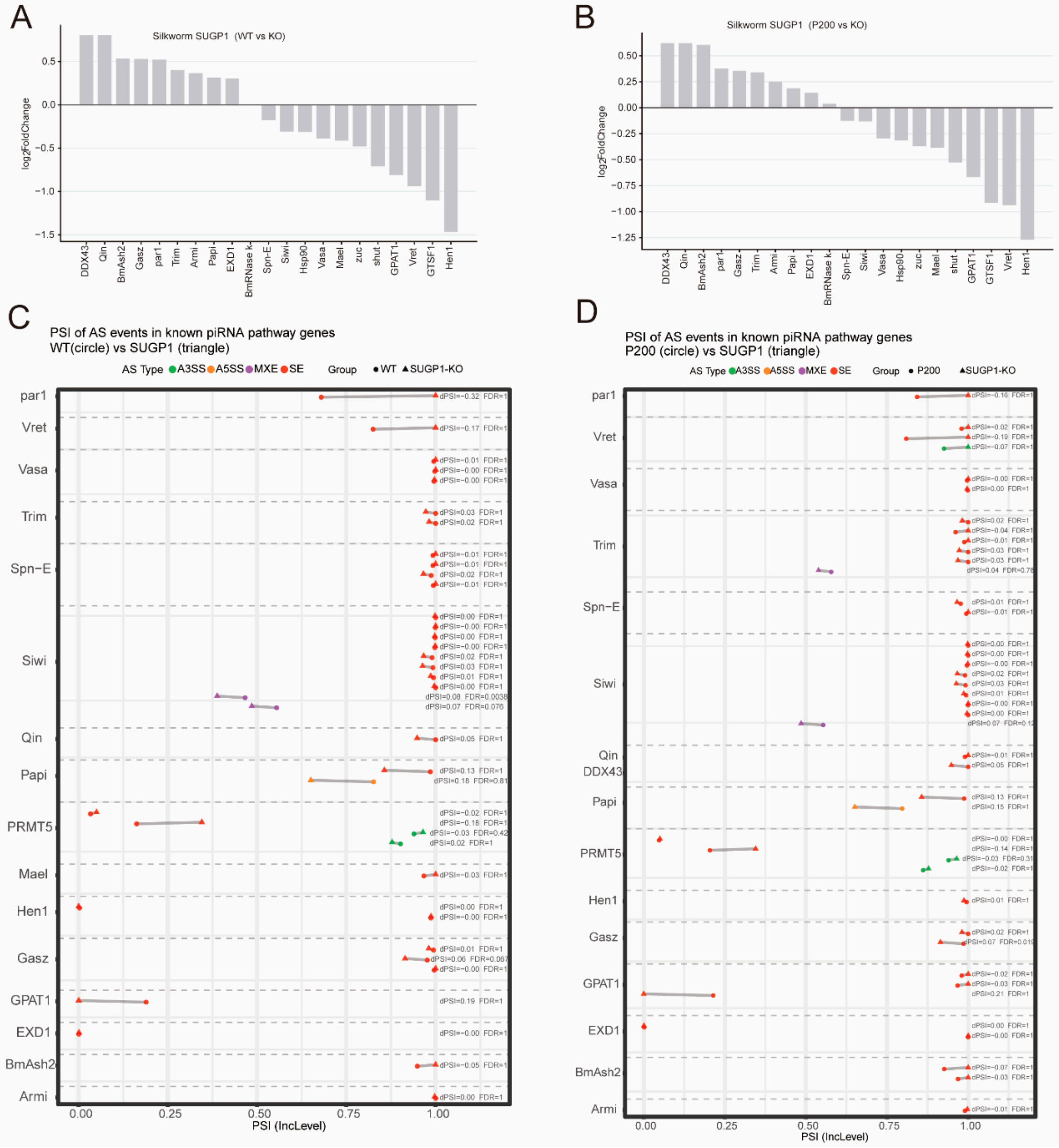

Figure S7. Expression and splicing profiles of known piRNA pathway genes after SUGP1 dysregulation with an independent sgRNA.

(A-B) RNA-seq analysis of expression changes in the known piRNA pathway components in *B. mori* SUGP1-KO polyclonal cell populations. The control is WT in (A) and empty vector (P200) in (B). (C-D) Alternative splicing profiles of known piRNA biogenesis pathway genes following

SUGP1 knockout. The control is WT in (C) and empty vector (P200) in (D).
