# Supplementary material for: SUGP1 Deficiency Leads to Splicing Dysregulation and Reduced piRNA Abundance in Insects
Source: Biology (Basel). 2026 Oct 15;15(20):1852. doi: 10.3390/biology15201852 (PMC13701588; doi:10.3390/biology15201852)
Supplement: Supplementary file 1 [file biology-15-01852-s001.zip › Supplementary File S2 Uncropped_original_western_blots.pdf]

Original Uncropped Western Blot Images

Manuscript ID: biology-4579761

File organization

For each figure, the original full-blot images containing explanatory annotations identifying molecular-weight positions, lane identities, antibodies, and expected target bands are presented first. The corresponding processed panel from the manuscript is then provided solely for cross-reference.

Figure 5A Original full-blot images

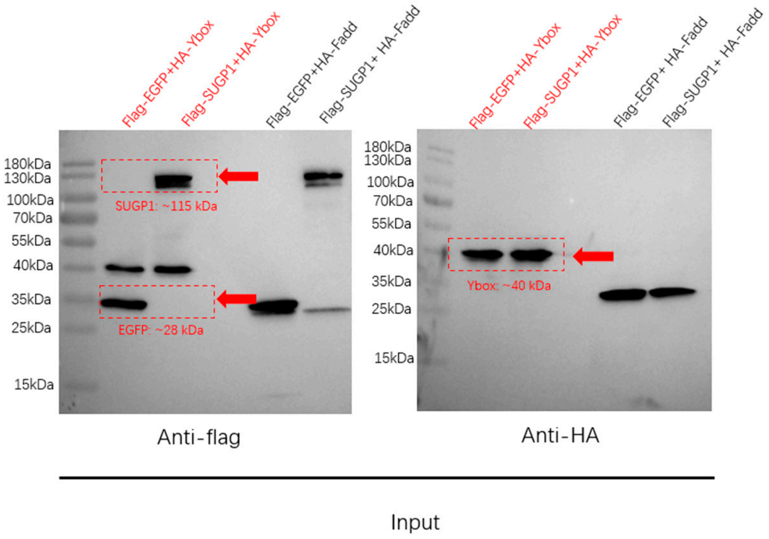

Figure 5A source image 1. Input samples probed with anti-Flag and anti-HA antibodies. Red labels, arrows, and dotted boxes are explanatory annotations in the supplied source image; they identify the relevant lanes and expected band positions.

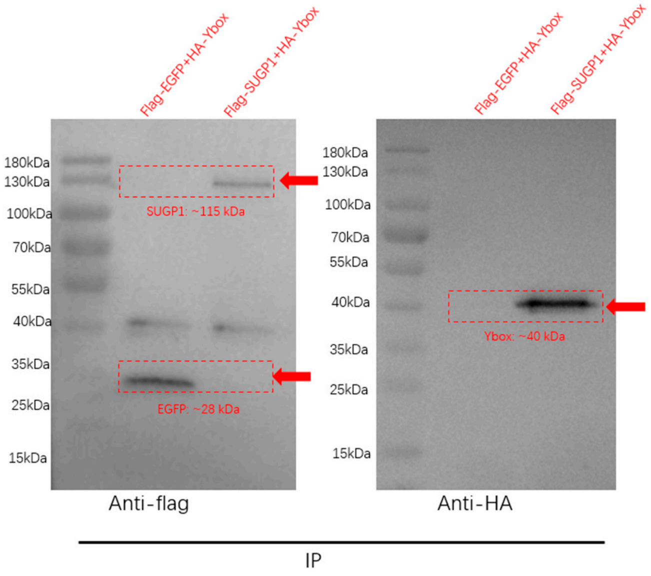

Figure 5A source image 2. Immunoprecipitation samples probed with anti-Flag and anti-HA antibodies.

Figure 5A Corresponding manuscript panel

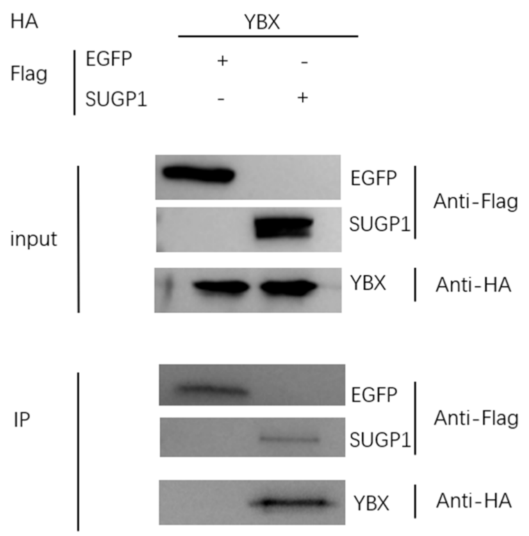

Corresponding processed panel used in Figure 5A of the manuscript.
This processed panel is included only to facilitate comparison with the original full-blot images on the preceding pages.

Figure 5B Original full-blot images

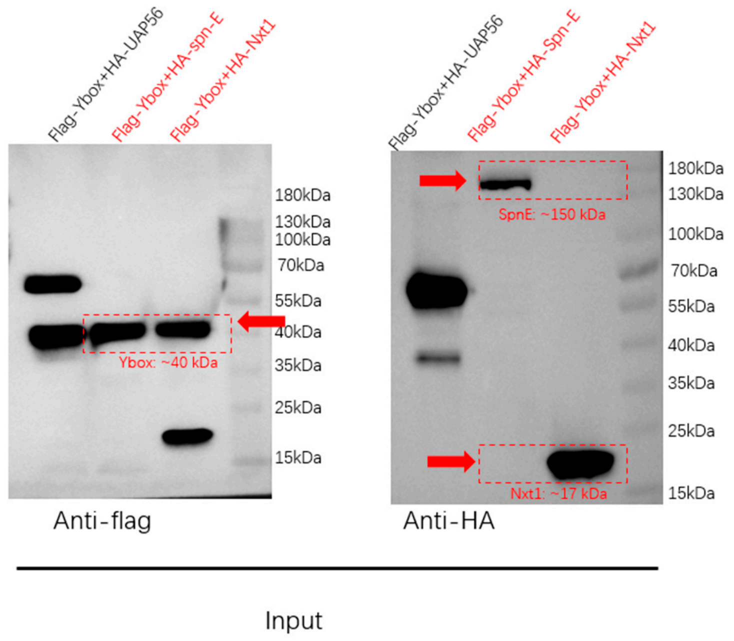

Figure 5B source image 1. Input samples probed with anti-Flag and anti-HA antibodies. The source image identifies the YBX, Spn-E, and Nxt1 expression constructs, molecular-weight positions, and expected target bands.

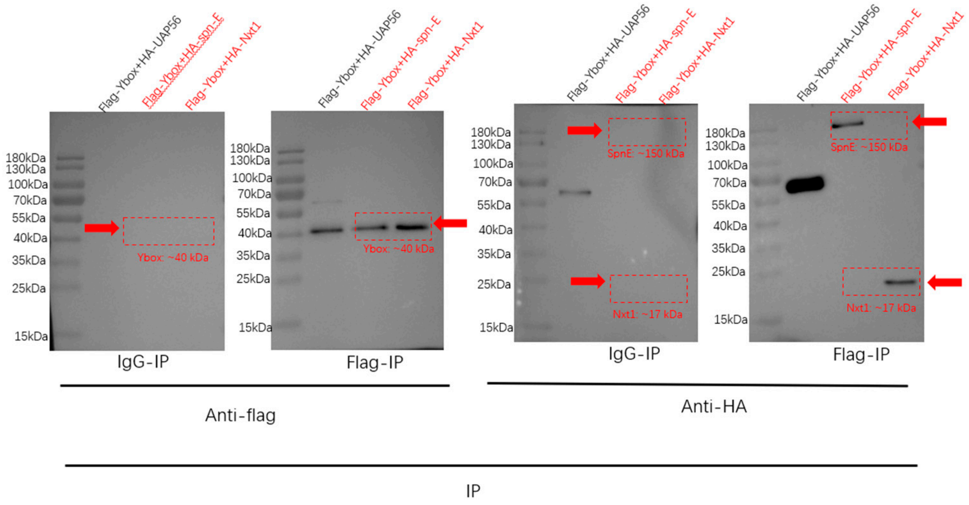

Figure 5B source image 2. IgG-IP and Flag-IP samples probed with anti-Flag and anti-HA antibodies.

Figure 5B Corresponding manuscript panel

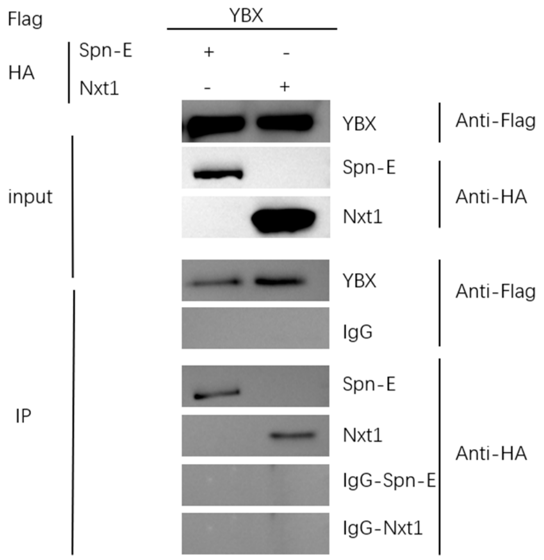

Corresponding processed panel used in Figure 5B of the manuscript.
This processed panel is included only to facilitate comparison with the original full-blot images on the preceding pages.

Notes:
The protein maker is indicated on both sides of the figure.
The lanes in red font are the target lanes.
The target bands are outlined by red dotted boxes.
The expected protein size is indicated by a red arrow along with a description of the expected size.
The presented picture is from the third replicate experiment.
