# Supplementary material for: SUGP1 Deficiency Leads to Splicing Dysregulation and Reduced piRNA Abundance in Insects
Source: Biology (Basel). 2026 Oct 15;15(20):1852. doi: 10.3390/biology15201852 (PMC13701588; doi:10.3390/biology15201852)
Supplement: Supplementary file 1 [file biology-15-01852-s001.zip › Supplementary File S3 Uncropped_original_gel_images.pdf]

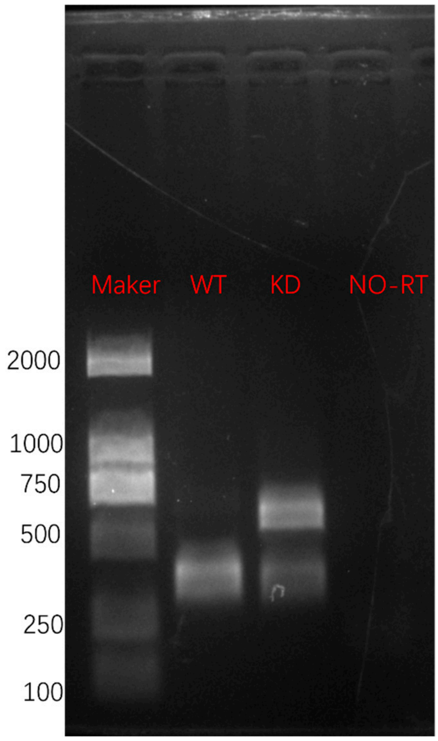

GeneID	geneSymbol	PValue	FDR	EventType
FBgn0027549	Nulp1	6.19E-05	0.0078	A3SS

Canonical splicing yields a 298 bp amplification product. Under SUGP1-KD conditions, aberrant splice site recognition (via A3SS) occurs, resulting in an abnormal transcript of more than 298 bp.

Primer Sequences

>Nulp1-F1(298)

AATCGCTCTTGCCTTCGTTT

>Nulp1-R1

CCCTCCTCCTCGTCGTTGCT

The results of other biological replicates

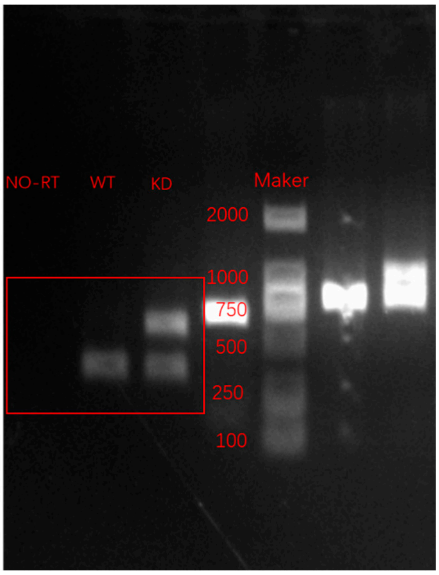
NO-RT
WT
KD
Maker
2000
1000
750
500
250
100
